# Supplementary figures and images for: Glycine-serine-rich effector PstGSRE4 in Puccinia striiformis f. sp. tritici inhibits the activity of copper zinc superoxide dismutase to modulate immunity in wheat
Source: PLoS Pathog. 2022 Jul 26;18(7):e1010702. doi: 10.1371/journal.ppat.1010702 (PMC9321418; doi:10.1371/journal.ppat.1010702)

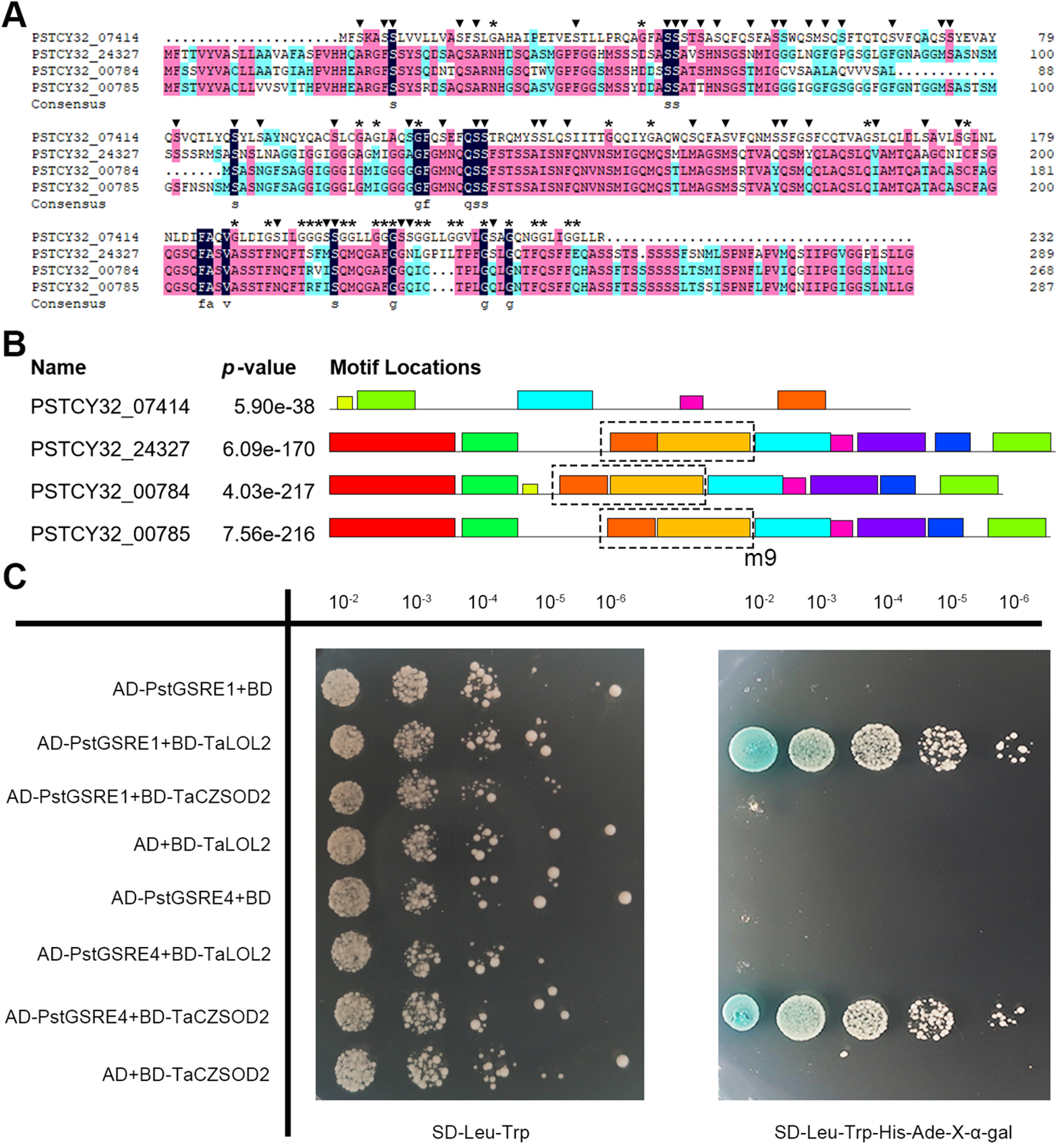

Supplement: S1 Fig — (A) Sequence analysis indicates that PstGSRE4 is a glycine- and serine-rich secreted protein. Triangles indicate serine and asterisks represent glycine residues. Multi-sequence alignment of PstGSRE4 and other three glycine- and serine-rich secreted proteins was performed using CLC Sequence Viewer. (B) The motif of PstGSRE4 (PSTCY32_07414) and other three glycine- and serine-rich secreted proteins were predicted by MEME suit (http://meme-suite.org/). The black borders represent m9 region of PstGSRE1 (PSTCY32_24327). (C) PstGSRE4 cannot interact with TaLOL2. Only the yeast co-expressing PstGSRE4 and TaCZSOD2 or PstGSRE1 and TaLOL2 grew on the medium SD-Trp-Leu-His-Ade and yielded X-α-gal activity. Yeast strains co-expressing PstGSRE4 and TaLOL2 or PstGSRE1 and TaCZSOD2 cannot grow on the medium SD-Trp-Leu-His-Ade. This experiment was repeated three times. (TIF) [file ppat.1010702.s001.tif]

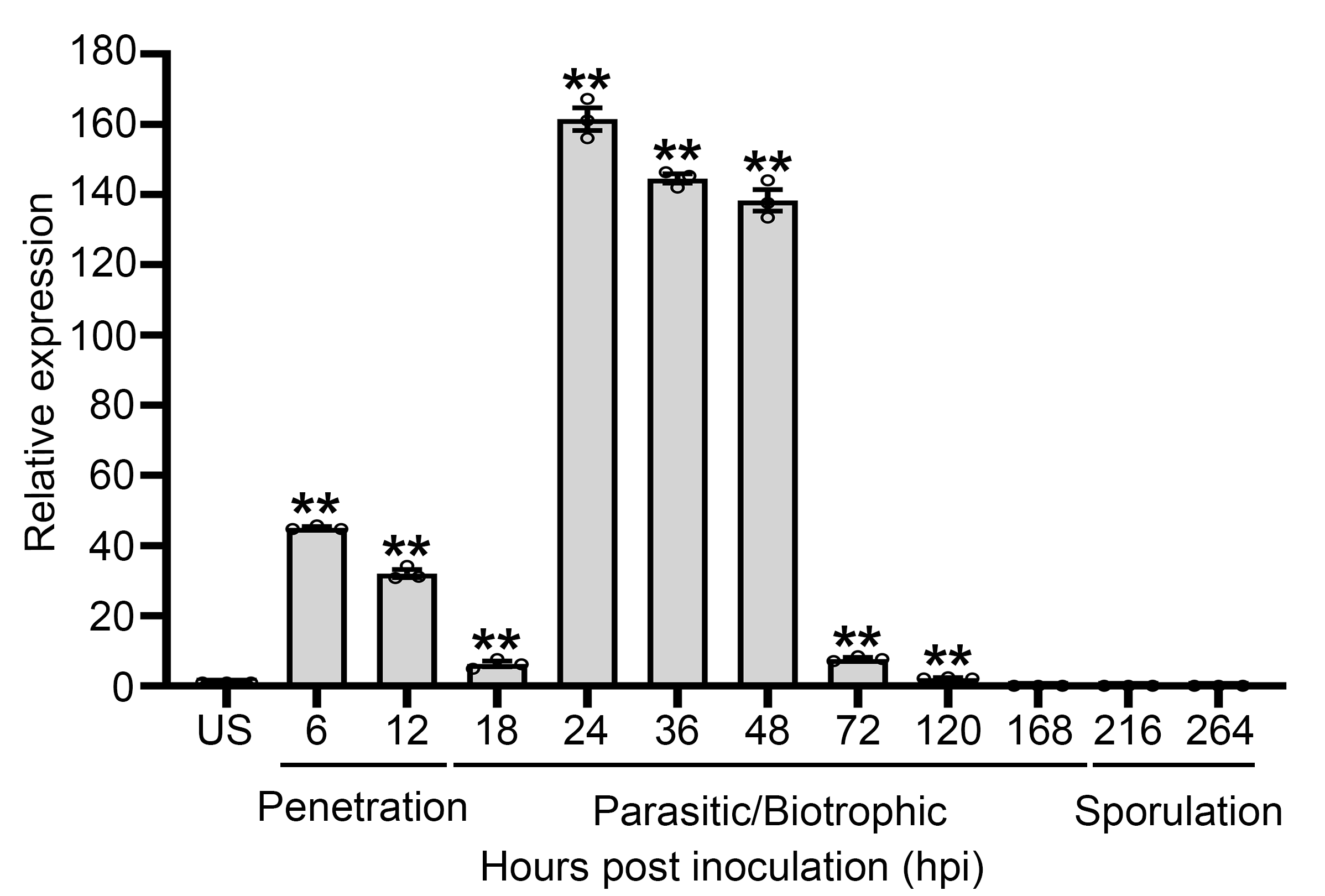

Supplement: S2 Fig — Wheat leaves (Suwon11) inoculated with freshly collected urediniospores (CYR32) were sampled at different time points according to the infection stage of Pst. US (Urediniospores) was used as a control. Relative transcript levels of PstGSRE4 were calculated by the comparative threshold (2-ΔΔCT) method. The quantitative RT-PCR values were normalized to the expression level for PstEF-1. The transcript level of PstGSRE4 at US stage was standardized as 1. Values represent the means ± SE of three independent replicates. Differences between time-course points were assessed using Student’s t-test. Double asterisks indicate P < 0.01. (TIF) [file ppat.1010702.s002.tif]

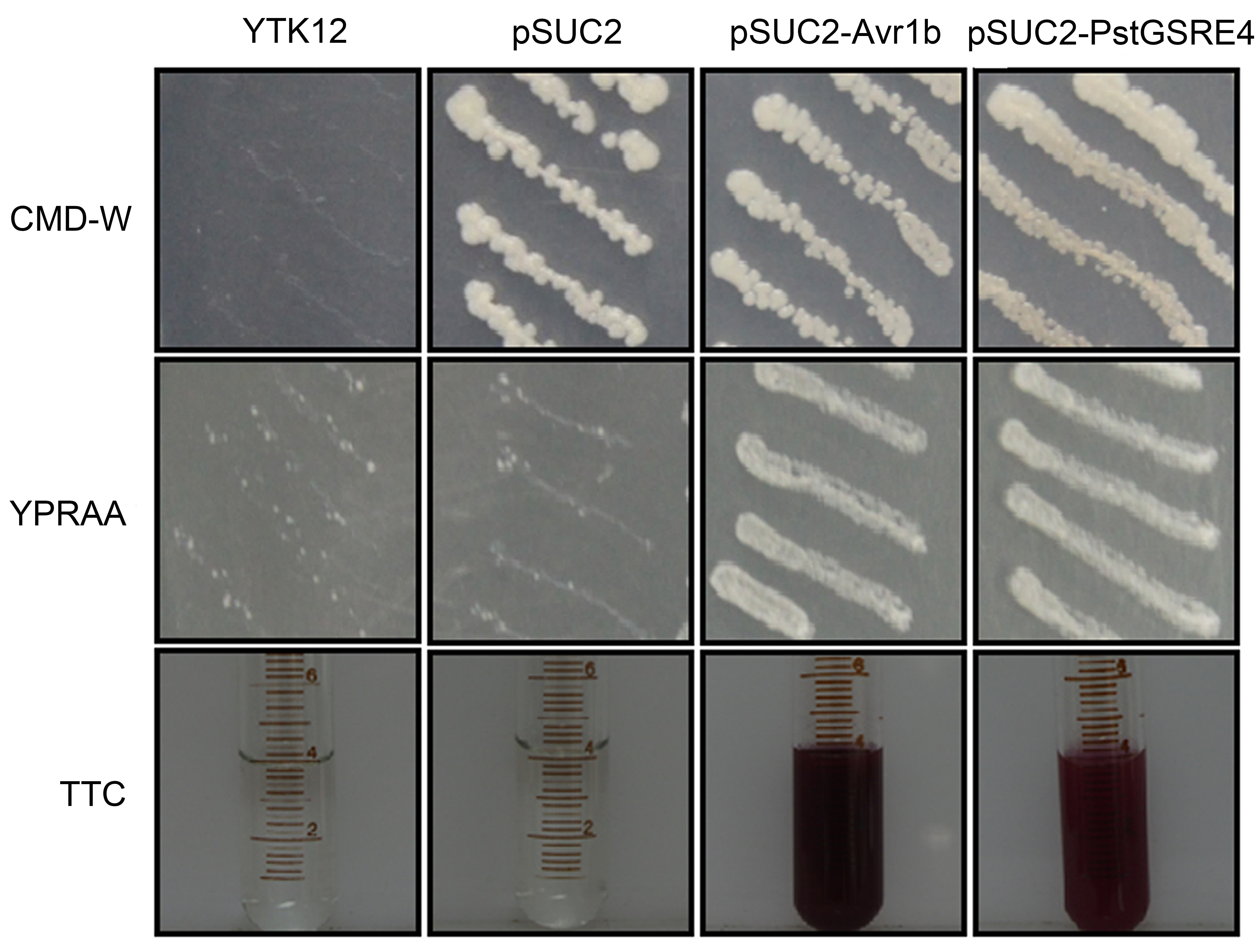

Supplement: S3 Fig — Functional validation of the putative N-terminal signal peptide of PstGSRE4 using the yeast invertase secretion assay. Yeast YTK12 strains carrying pSUC2-SP (Avr1b) and pSUC2-SP (PstGSRE4), which express two different signal peptides fused in frame to the mature invertase gene SUC2, were able to grow in YPRAA (Yeast-Peptone-Raffinose-Antimycin A) medium with raffinose as sole carbon source. YTK12 or YTK12 strains carrying empty vector pSUC2T7M13ORI were used as negative control. Invertase activity was detected with 2,3,5-triphenyltetrazolium chloride (TTC). The red color indicates invertase activity. (TIF) [file ppat.1010702.s003.tif]

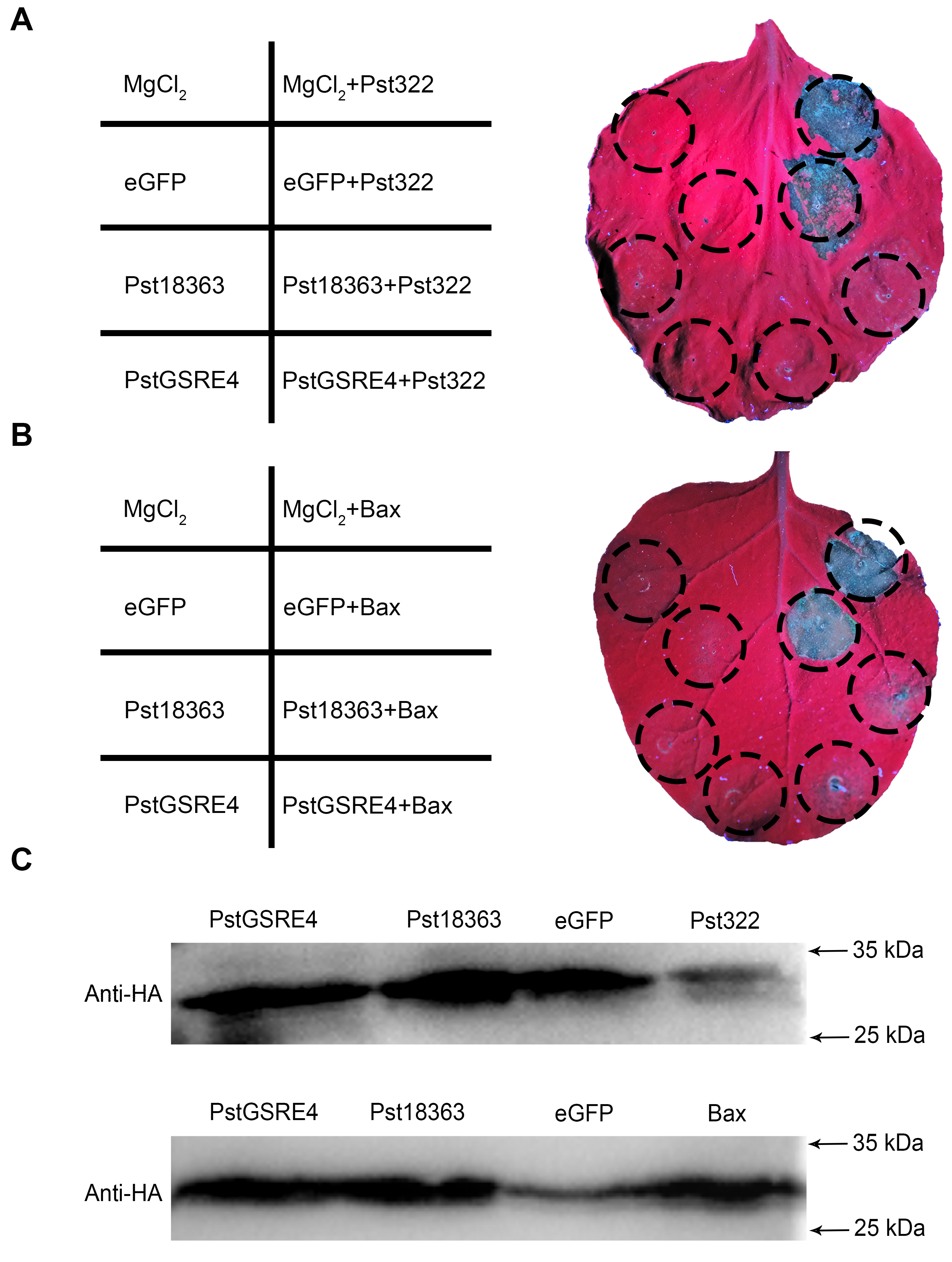

Supplement: S4 Fig — (A) PstGSRE4 suppressed Pst322-induced cell death. Photos of N. benthamiana leaves were taken under ultraviolet light. (B) PstGSRE4 suppressed Bax-induced cell death. Photos of N. benthamiana leaves were taken under ultraviolet light. (C) Western blot with anti-HA antibody was performed to show normal expression of eGFP-HA (25kDa), Pst18363-HA (20kDa), PstGSRE4-HA (22kDa), Pst322-HA (20kDa) and Bax-HA (25kDa) in tobacco leaves. (TIF) [file ppat.1010702.s004.tif]

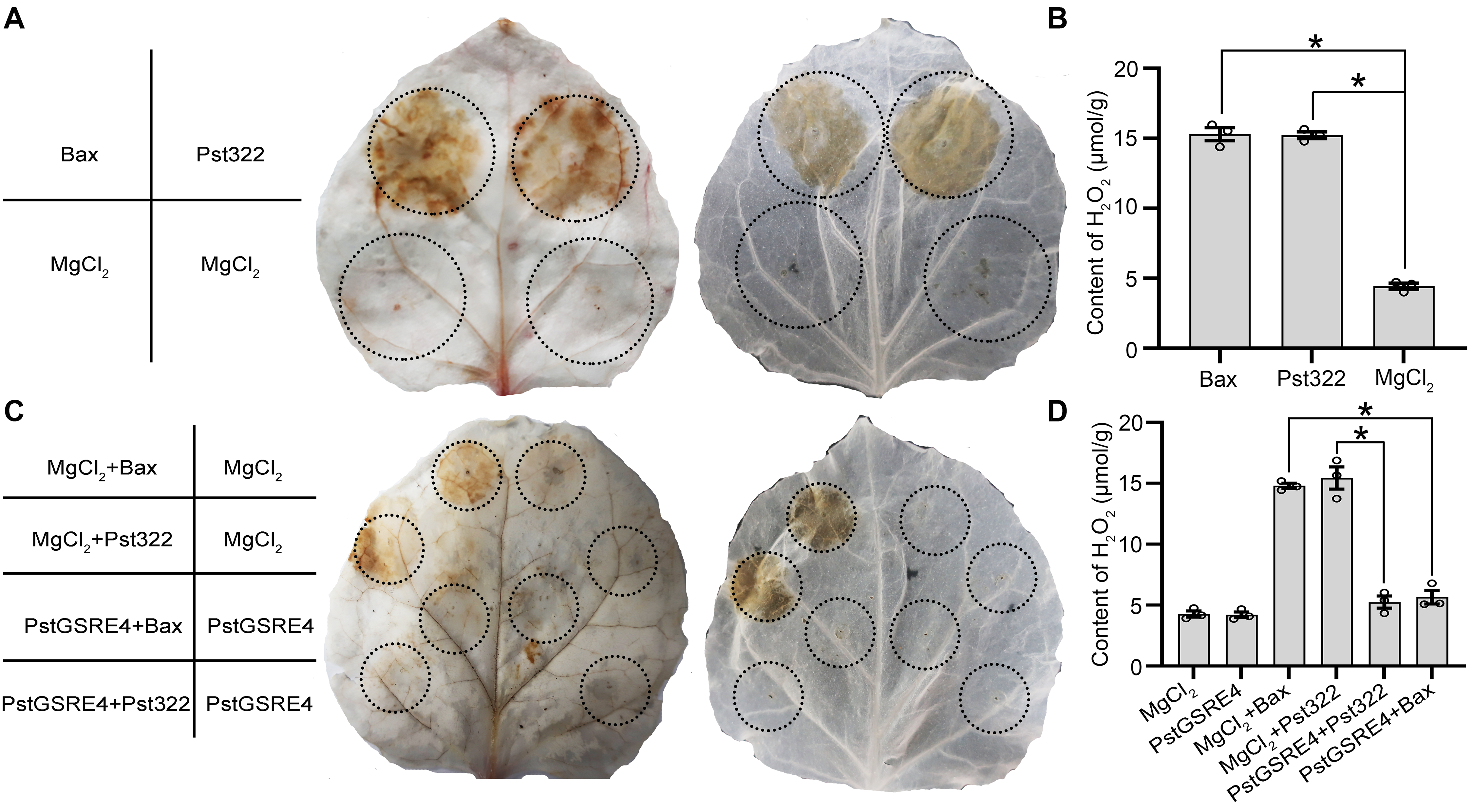

Supplement: S5 Fig — (A) H2O2 production in N. benthamiana leaves was determined by DAB staining. The measurement was performed at 3 d after infiltration with Pst322 or Bax (left). And overexpression of Bax or Pst322 in Nicotiana benthamiana triggered programmed cell death (PCD) at 4 d after infiltration with Pst322 or Bax (right). (B) Content of H2O2 in N. benthamiana leaves was determined at 3 d after infiltration with Pst322 or Bax. Values represent the means ± SE of three independent samples. (C) H2O2 production in N. benthamiana leaves was determined by DAB staining. N. benthamiana leaves were infiltrated with A. tumefaciens cells carrying the PstGSRE4-HA or MgCl2 buffer, followed after 24 h by infiltration with A. tumefaciens cells carrying the Bax or Pst322. The measurement was performed at 3 d after infiltration with Bax or Pst322 (left). Overexpression of PstGSRE4 in Nicotiana benthamiana suppressed programmed cell death (PCD) triggered by Bax or Pst322 at 4 d (right). (D) Content of H2O2 in N. benthamiana leaves was determined at 3 d after infiltration with PstGSRE4, MgCl2, PstGSRE4/Bax, PstGSRE4/Pst322, MgCl2/Bax or MgCl2/Pst322. Values represent the means ± SE of three independent samples. (TIF) [file ppat.1010702.s005.tif]

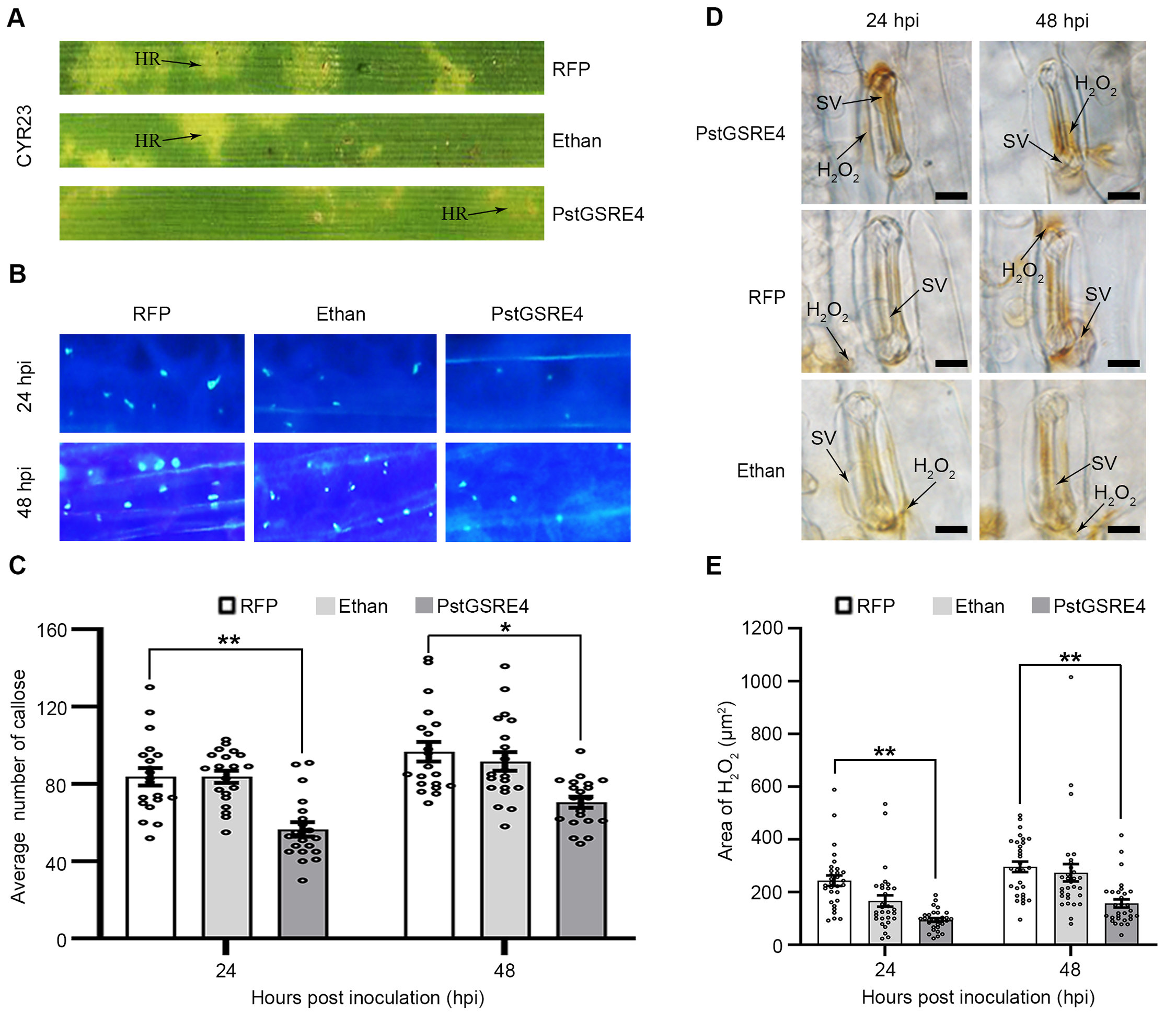

Supplement: S6 Fig — (A) Phenotypes of wheat leaves (Suwon11) inoculated with CYR23 after being injected with Pseudomonas fluorescens strain EtHAn alone or carrying plasmids pEDV6-RFP (a red autofluorescent protein, DsRed), or pEDV6-PstGSRE4 at 14 dpi. HR, hypersensitive response. (B-C) Wheat leaves inoculated as above were examined for callose deposition by epifluorescence microscopy after aniline blue staining. Scale bars, 100 μm. The average number of callose deposits per mm2 at 24 and 48 hpi was counted using ImageJ software. Values represent the means ± SE (n = 20). (D-E) H2O2 production in leaves infiltrated with EtHAn, EtHAn pEDV6-RFP, or EtHAn pEDV6-PstGSRE4 at 24 and 48 hpi with Pst. SV, substomatal vesicle. Tissues were stained with DAB. Scale bars, 20 μm. The amount of H2O2 production was measured by calculating the DAB-stained area at each infection site using DP-BSW software. Values represent the means ± SE (n = 30). Asterisks indicate a significant difference (P < 0.05) relative to the control sample according to Student’s t-test, double asterisks indicate P < 0.01. (TIF) [file ppat.1010702.s006.tif]

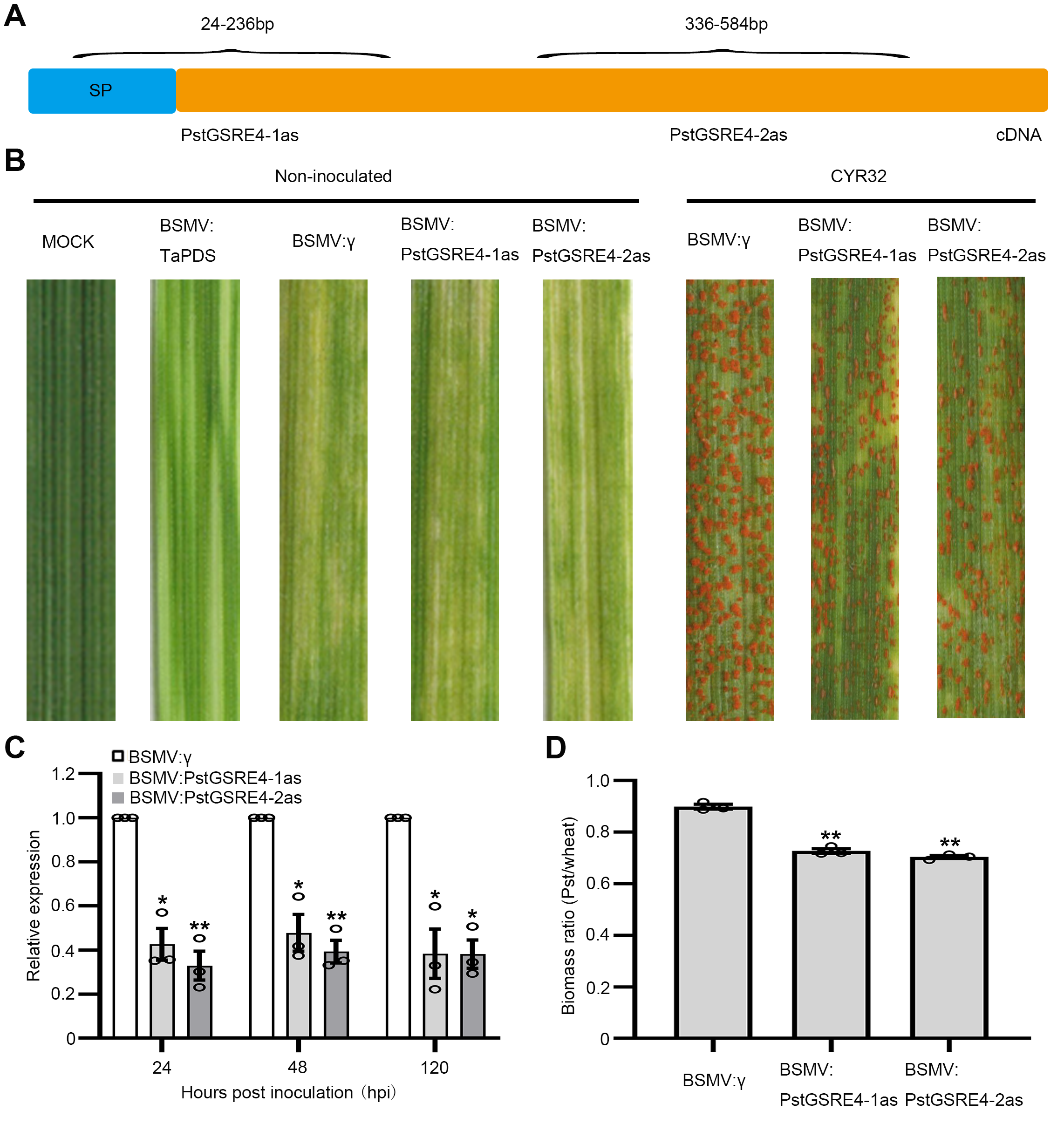

Supplement: S7 Fig — (A) Two specific sequence regions were selected for BSMV-mediated transient silencing. (B) Wheat leaves infected with BSMV:TaPDS, which showed photobleaching phenotype, was used as control. Mild chlorotic mosaic symptoms were observed on the fourth leaves of the wheat inoculated with BSMV:γ, BSMV:PstGSRE4-1as, and BSMV:PstGSRE4-2as. Phenotypes of the fourth leaves of knockdown plants or control plants inoculated with Pst race CYR32 at 12 dpi. (C) Relative transcript levels of PstGSRE4 in PstGSRE4-knockdown plants challenged by CYR32. PstEF-1 was used for normalization. Values represent the means ± SE (n = 3). (D) Ratio of fungal to wheat nuclear content using fungal PstEF-1 and wheat TaEF-1α genes, respectively. Genomic DNA was extracted from the second leaf from three different plants at 14 dpi. Values represent the means ± SE (n = 3). Differences were assessed using Student’s t-test, and asterisks indicate P < 0.05, double asterisks indicate P < 0.01. (TIF) [file ppat.1010702.s007.tif]

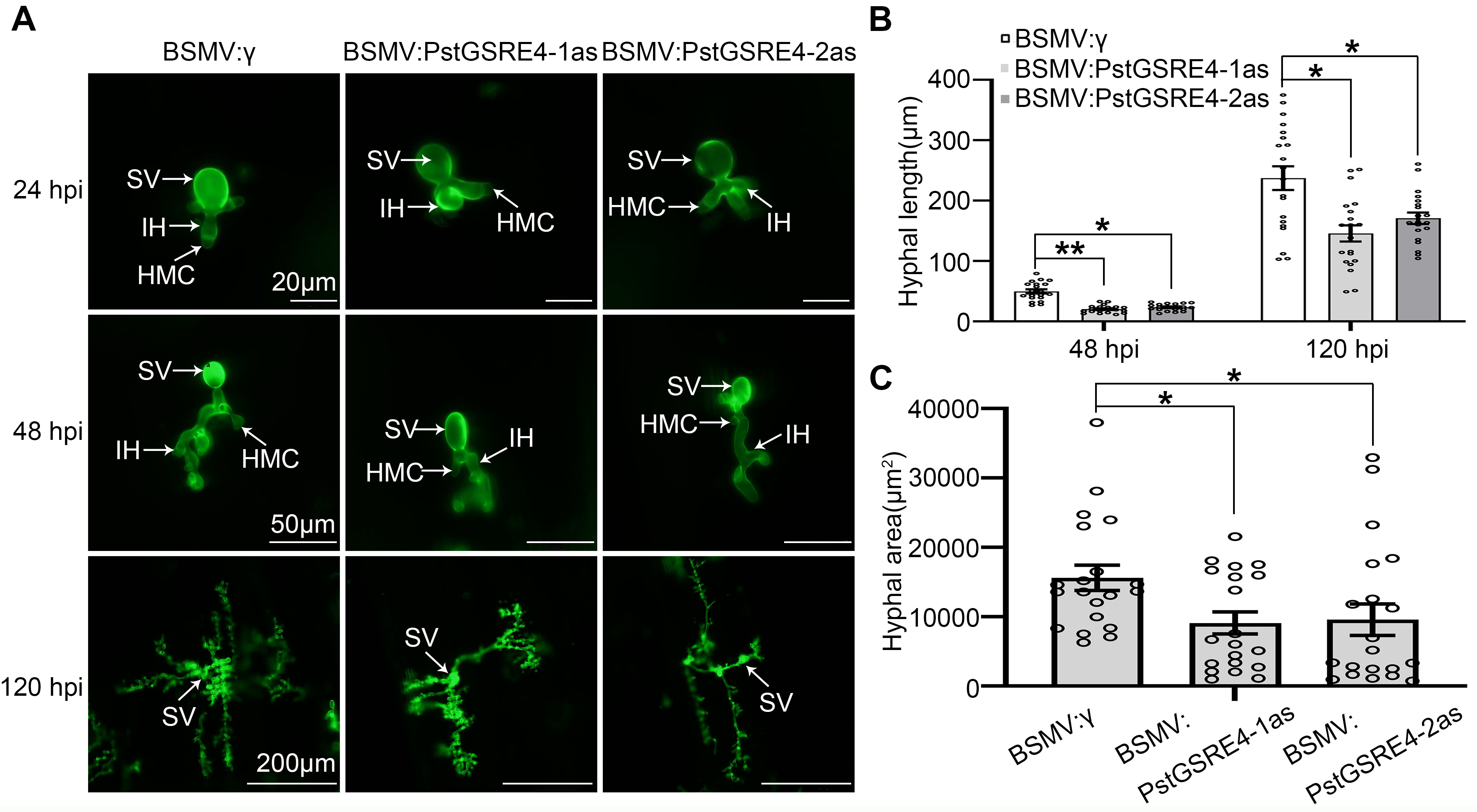

Supplement: S8 Fig — (A) Fungal growth at 24, 48 and 120 hpi in wheat leaves inoculated with BSMV:γ, BSMV:PstGSRE4-1as and BSMV:PstGSRE4-2as. (B-C) Hyphal lengths (48 and 120 hpi) and colony sizes (120 hpi) in PstGSRE4-knockdown plants were stained with WGA and quantified with DP-BSW software. SV, substomatal vesicle. HMC, haustorial mother cell. IH, infection hypha. Values represent the means ± SE (n = 30, n = 20). Differences were assessed using Student’s t-test, and asterisks indicate P < 0.05. (TIF) [file ppat.1010702.s008.tif]

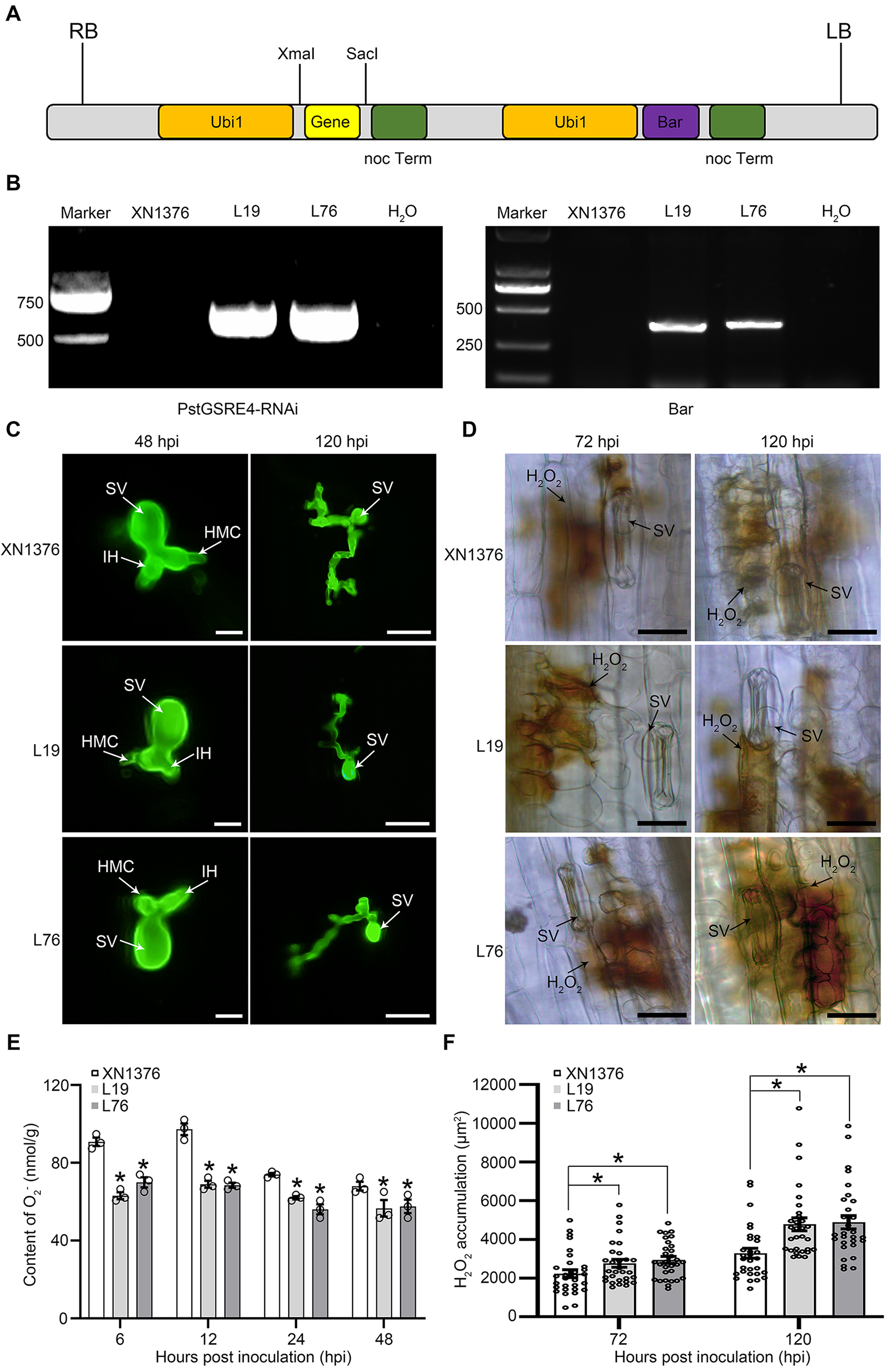

Supplement: S9 Fig — (A) Diagram showing the RNAi cassette in the wheat transformation construct pAHC25-PstGSRE4-RNAi. Ubi1, maize ubiquitin1 promoter. Adh1, Zea mays alcohol dehydrogenase 1. Noc Term, Nos terminator. Bar, Biolaphos resistance gene. LB, left border. RB, right border. (B) Transgenic plants were analyzed by genomic PCR for the presence of the selectable marker Bar gene and the fragment of the RNAi cassette (PstGSRE4). (C) Leaves inoculated with Pst race CYR31 were sampled at 48 and 120 hpi and examined under epifluorescence after staining with WGA conjugated to Alexa-488. SV, substomatal vesicle. HMC, haustorial mother cell. IH, infection hypha. Scale bars, 20 μm (left) and 50 μm (right). (D) H2O2 accumulation was measured in transgenic plants at 72 and 120 hpi. DAB was used to detect H2O2 viewed under differential interference contrast optics. Scale bars, 20 μm. (E) Content of O2− accumulation in different transgene lines at 6, 12, 24 and 48 hpi. Values represent the means ± SE of three independent samples. (F) Quantification of H2O2 accumulation in different transgenic lines at 72 and 120 hpi. Values represent the means ± SE (n = 30). Differences were assessed using Student’s t-test. Asterisk indicates P < 0.05. (TIF) [file ppat.1010702.s009.tif]

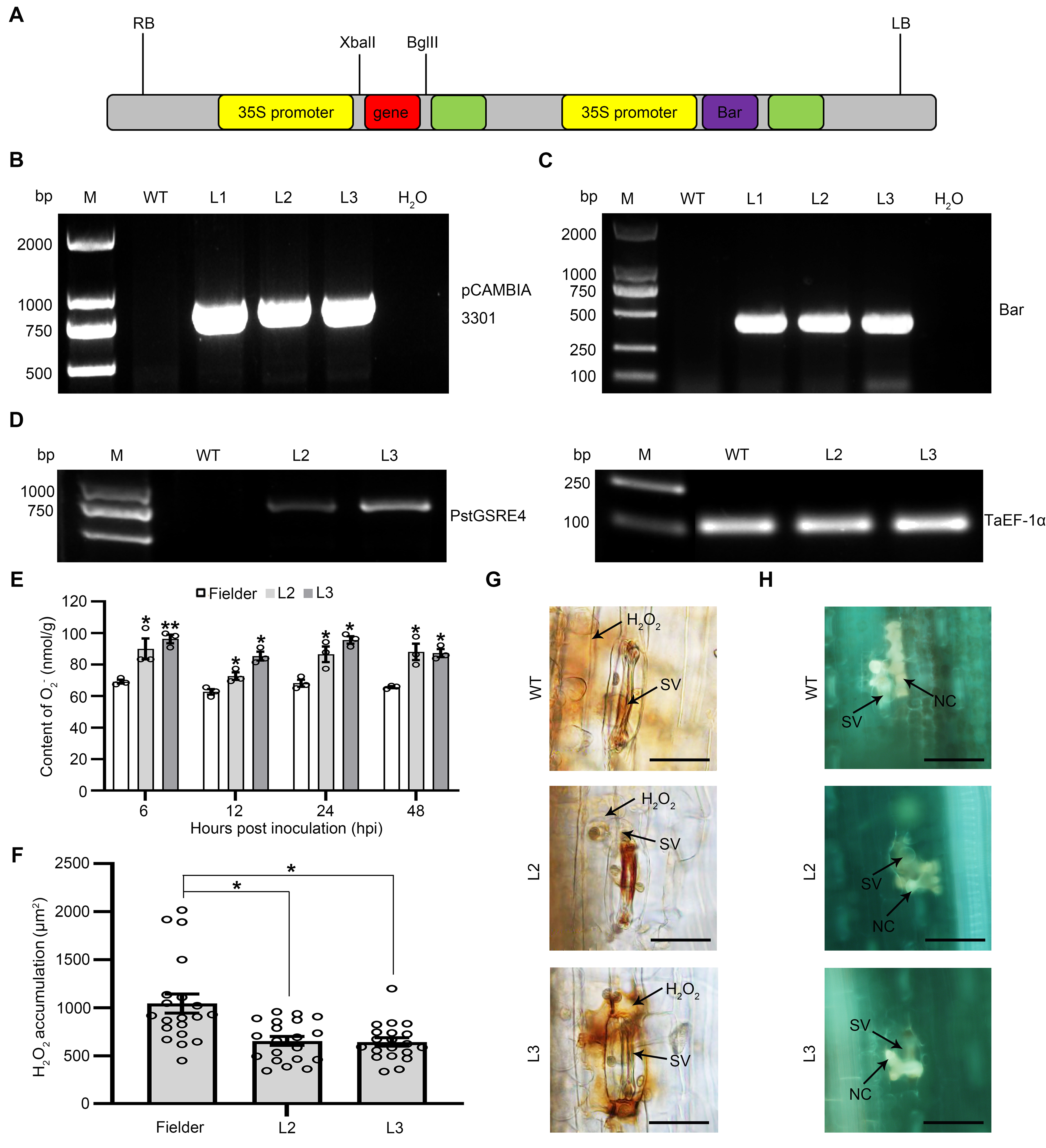

Supplement: S10 Fig — (A) Diagram showing the overexpression cassette in the wheat transformation construct pCAMBIA3301-PstGSRE4-overexpression. LB, left border. RB, right border. (B-C) Transgenic plants were analyzed by genomic PCR for the presence of the selectable marker Bar gene and the pCAMBIA3301 primer. (D) Expression of PstGSRE4 in T3 lines (L2 and L3) was analyzed by RT-PCR. Expression of TaEF-1α showed equal loading. (E) Content of O2- in different transgenic lines at 6, 12, 24 and 48 hpi. Values represent the means ± SE of three independent samples. (F) Quantification of H2O2 accumulation in different transgenic lines at 48 hpi. (G) H2O2 accumulation at infection sites were observed by microscopy after DAB staining. SV, substomatal vesicle. Scale bars, 20 μm. Values represent the means ± SE (n = 20). (H) Observation of necrotic cell death by epifluorescence in transgenic plants. NC, necrotic cell death. SV, substomatal vesicle. Scale bars, 20 μm. Differences were assessed using Student’s t-test, and asterisks indicate P < 0.05, double asterisks indicate P < 0.01. (TIF) [file ppat.1010702.s010.tif]

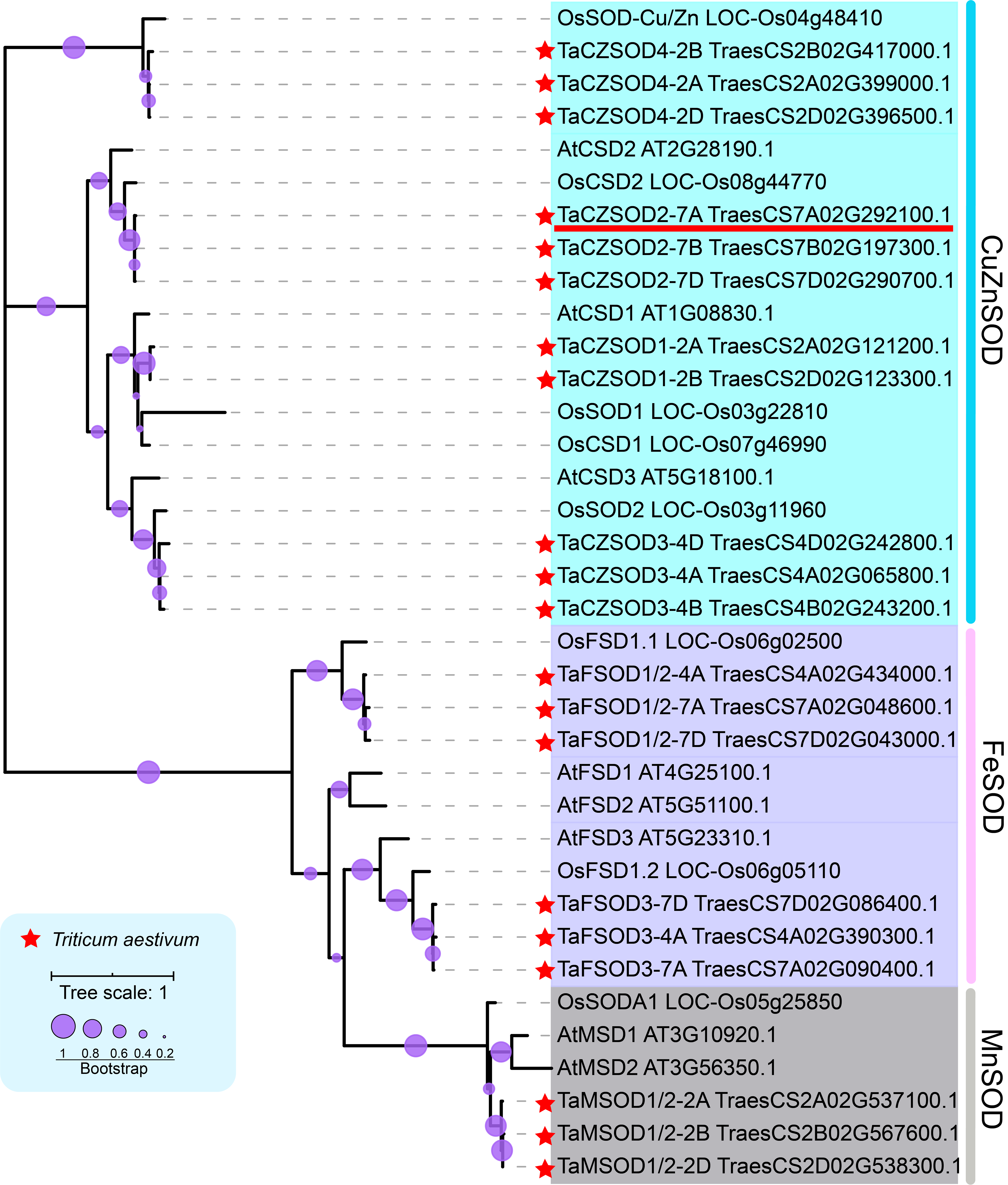

Supplement: S11 Fig — Branches are labeled with protein names and GenBank accession number. Os, Oryza sativa. At, Arabidopsis thaliana. The red line represents TaCZSOD2-7A. The tree was created with bootstrap of 1000 by maximum likelihood method in MEGA6. And the tree was drawn using Interactive Tree of Life (IToL). (TIF) [file ppat.1010702.s011.tif]

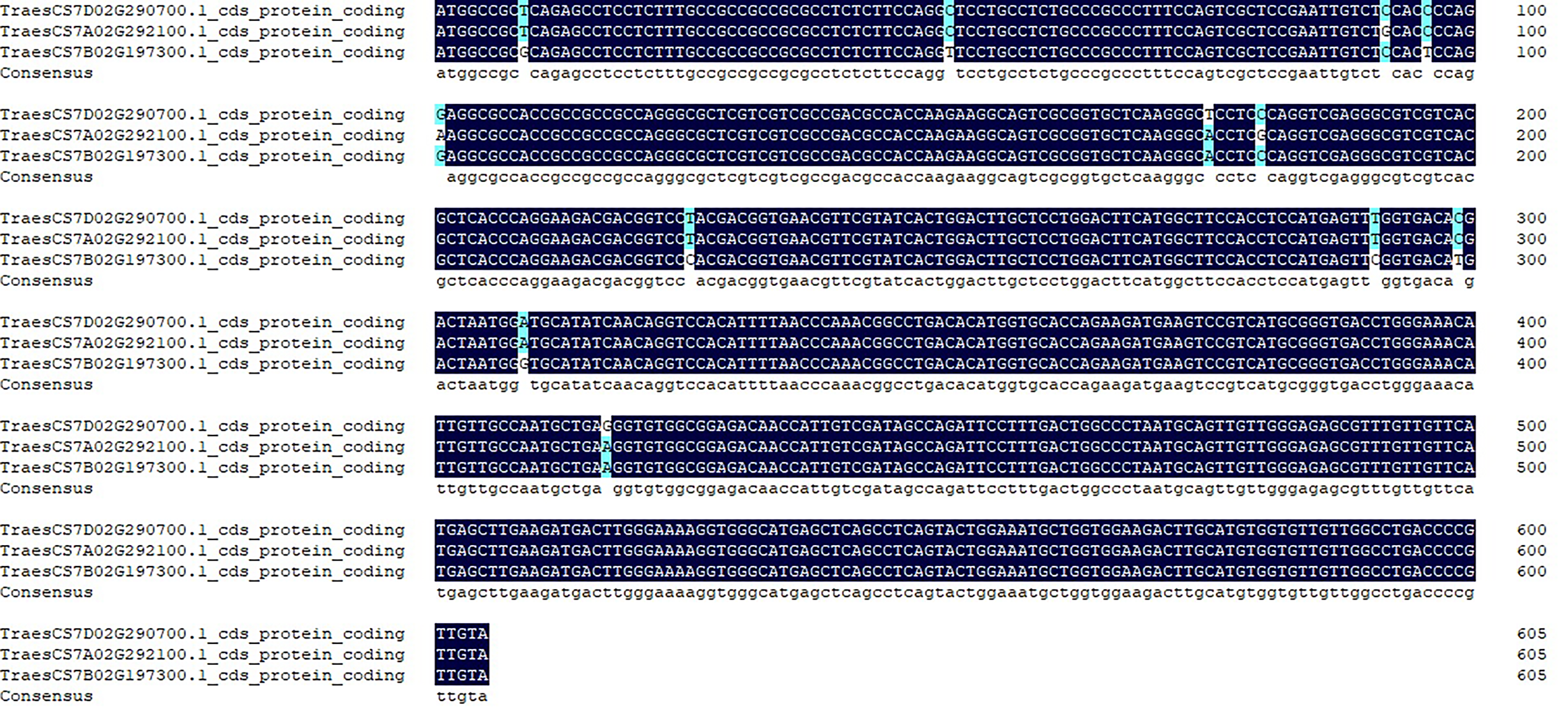

Supplement: S12 Fig — Multi-alignment of the coding sequences of the three copies of TaCZSOD2 in the genome database of wheat cultivar Chinese Spring. (TIF) [file ppat.1010702.s012.tif]

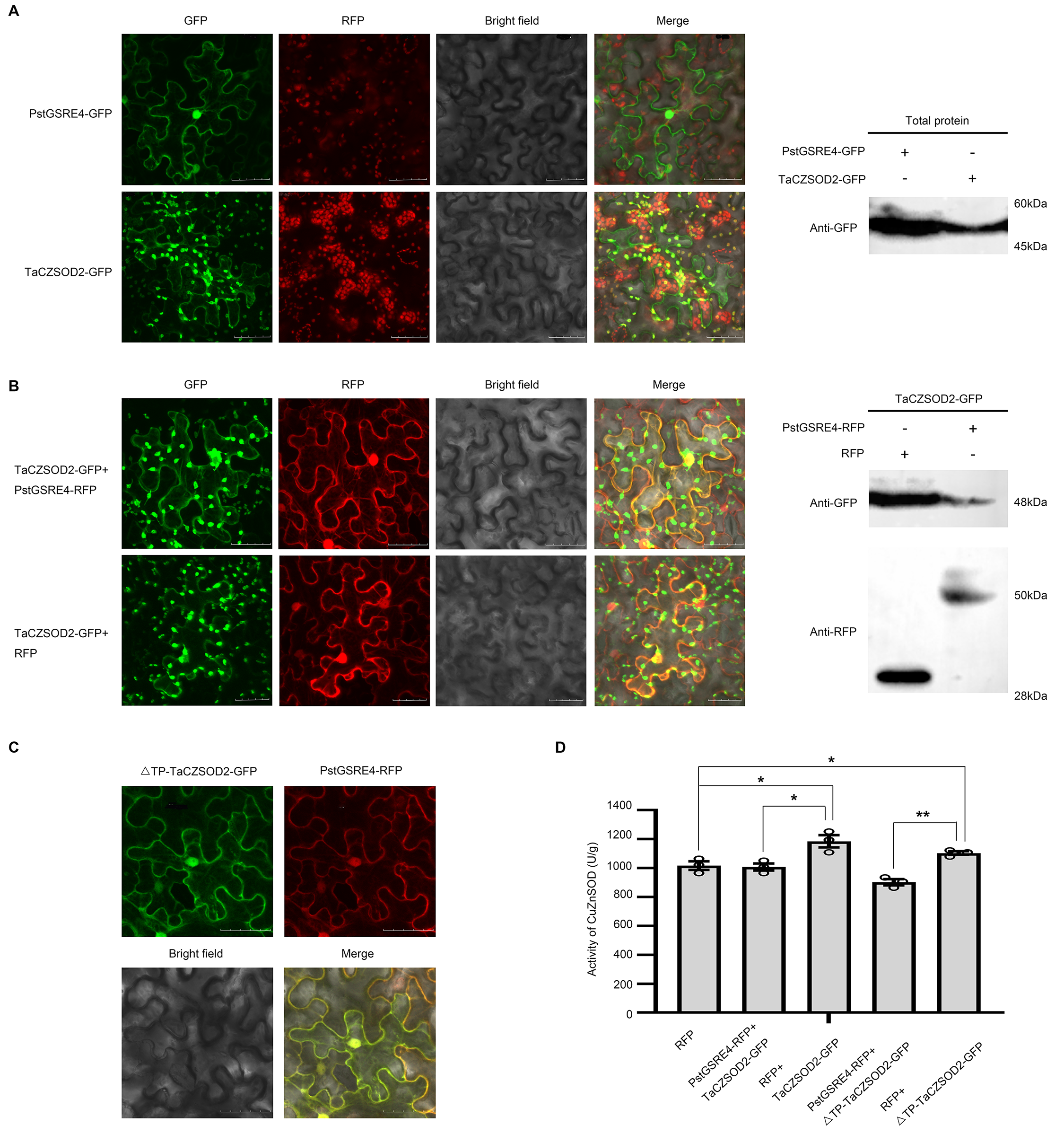

Supplement: S13 Fig — (A) Confocal microscopy images showing the subcellular localization of PstGSRE4 and TaCZSOD2. Western blotting analysis shows the total protein of PstGSRE4-GFP and TaCZSOD2-GFP in N. benthamiana leaves. Scale bars, 50 μm. (B) Co-localization of PstGSRE4-RFP (48 kDa) and TaCZSOD2-GFP (47 kDa) in N. benthamiana. In all panels, proteins were expressed in N. benthamiana through agroinfiltration. Fluorescence was detected in epidermal cells of the infiltrated leaves by confocal microscopy at 48 h after agroinfiltration (prior to any cell death). Scale bars, 50 μm. Localization of RFP-fusion of PstGSRE4 was uniformly located in the cytoplasm and nucleus, GFP-fusion of TaCZSOD2 was primarily located in the chloroplast, but also in the cytoplasm. When expressed together, co-localization of PstGSRE4 and TaCZSOD2 accumulated in the cytoplasm. Western blotting analysis shows the total protein of PstGSRE4-RFP and TaCZSOD2-GFP in N. benthamiana leaves. (C) Co-localization of GFP fusion of chloroplast transit peptide-deleted TaCZSOD2 (ΔTP-TaCZSOD2) and RFP-fusion of PstGSRE4 in N. benthamiana. They accumulated in the cytoplasm and nucleus. Scale bars, 50 μm. (D) Co-expressed GFP fusion of ΔTP-TaCZSOD2 with RFP-fusion of PstGSRE4, GFP fusion of TaCZSOD2 with RFP-fusion of PstGSRE4, GFP fusion of ΔTP-TaCZSOD2 with RFP, GFP fusion of TaCZSOD2 with RFP in N. benthamiana, and detected the activity of CuZnSOD. RFP was expressed as control. Values represent the means ± SE of three independent samples. These experiments were repeated three times and obtained the similar result. (TIF) [file ppat.1010702.s013.tif]

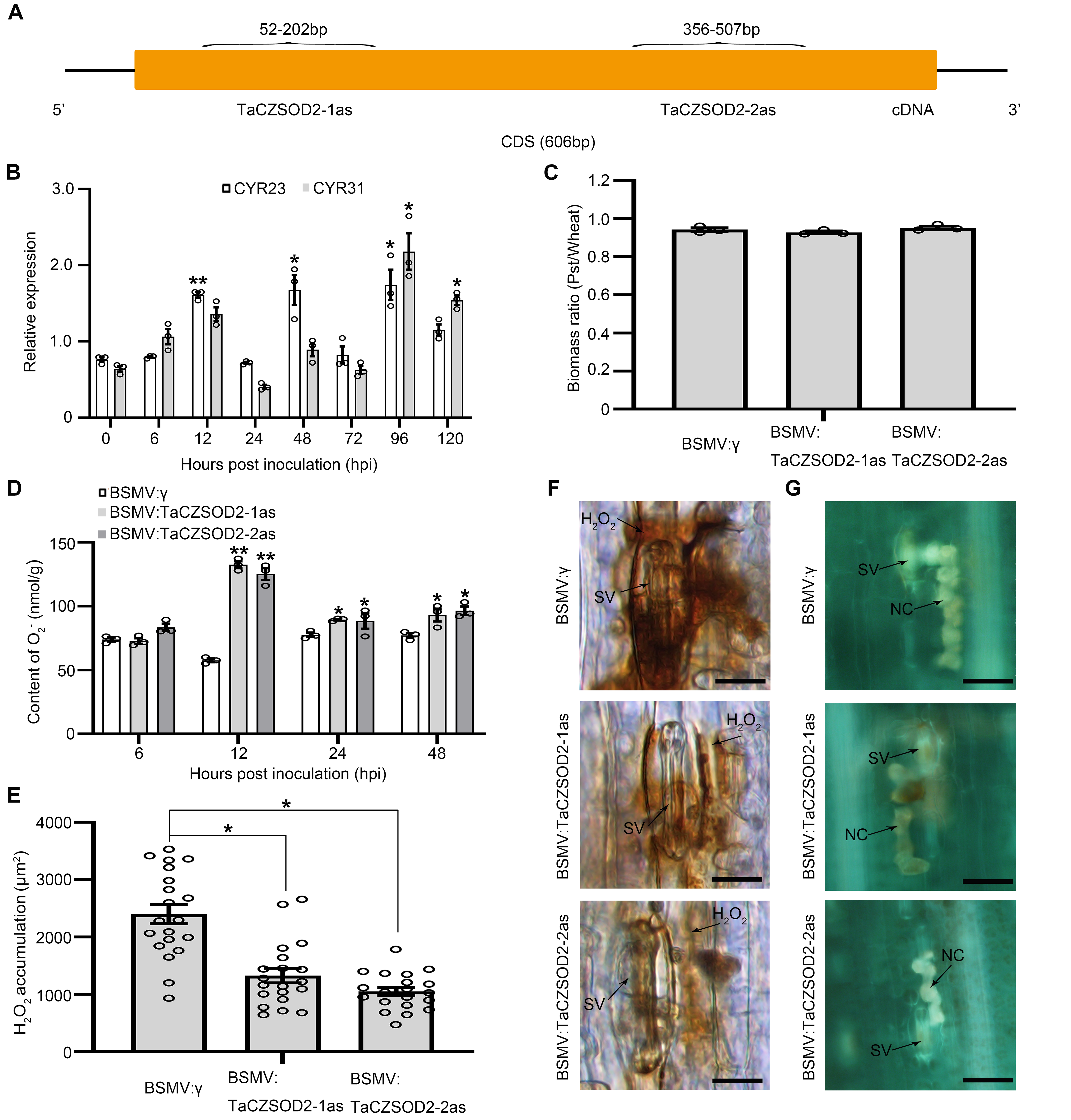

Supplement: S14 Fig — (A) Two specific sequence regions were selected for BSMV-mediated transient silencing. (B) Wheat leaves (Suwon11) inoculated with freshly collected urediniospores (CYR23 and CYR31) were sampled at different time points according to the infection stage of Pst. Relative transcript levels of TaCZSOD2 were calculated by the comparative threshold (2-ΔΔCT) method. The quantitative qRT-PCR values were normalized to the expression level for TaEF-1α. Differences between time-course points were assessed using Student’s t-tests. Asterisks indicate P < 0.05, double asterisks indicate P < 0.01. Values represent the means ± SE (n = 3). (C) After inoculated with CYR31, ratio of fungal to wheat nuclear content using fungal PstEF-1 and wheat TaEF-1α genes, respectively. Values represent the means ± SE (n = 3). (D) Content of O2− accumulation in TaCZSOD2-knockdown plants at 6, 12, 24 and 48 hpi. Values represent the means ± SE of three independent samples. (E) Quantification of H2O2 accumulation in TaCZSOD2-knockdown plants at 48 hpi. Values represent the means ± SE (n = 20). (F) H2O2 accumulation at infection sites was observed by microscopy after DAB staining. SV, substomatal vesicle. Scale bars, 10 μm. (G) Observation of necrotic cell death by epifluorescence in TaCZSOD2-knockdown wheat plants. NC, necrotic cell death. SV, substomatal vesicle. Scale bars, 20 μm. (TIF) [file ppat.1010702.s014.tif]

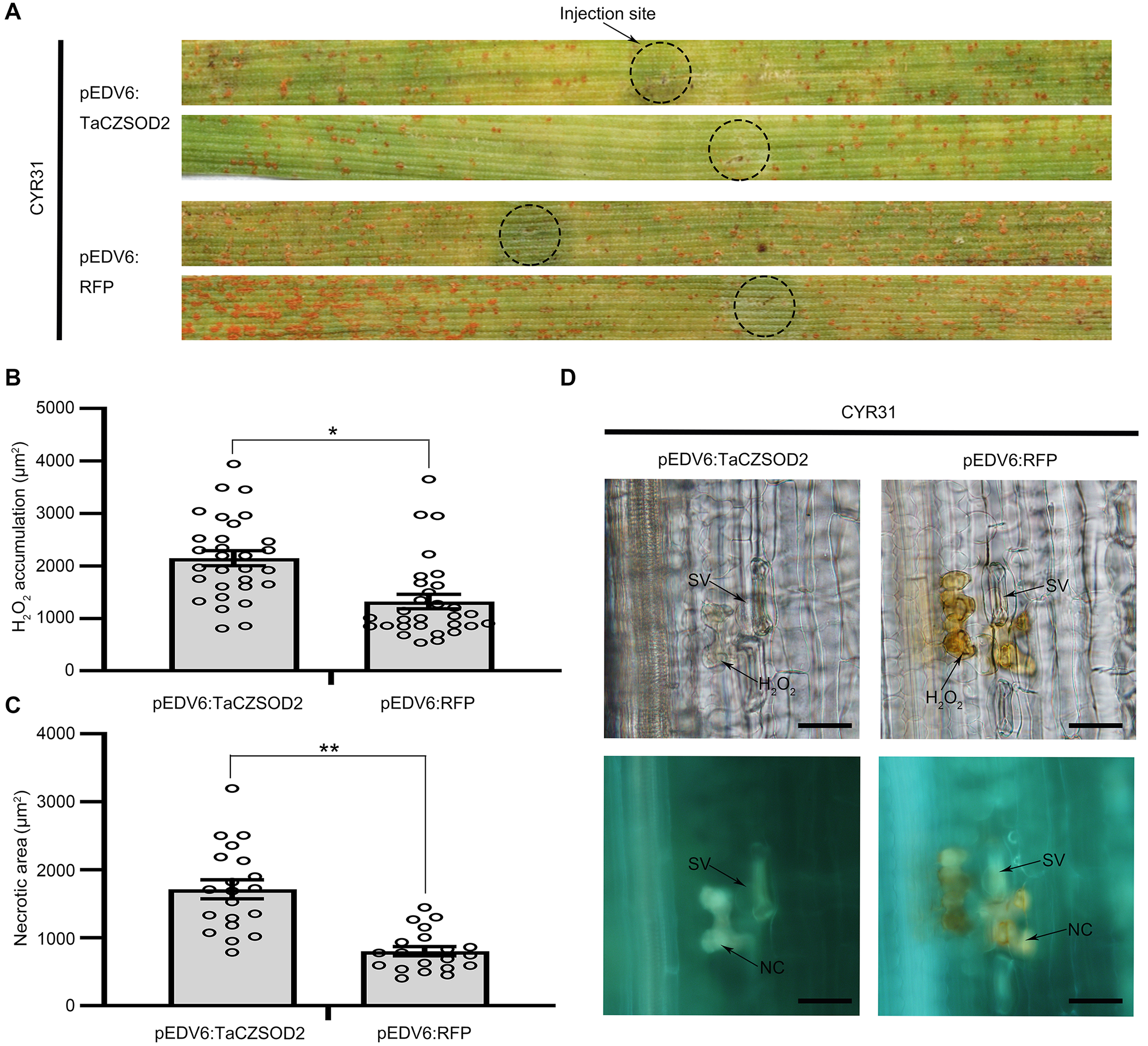

Supplement: S15 Fig — (A) Phenotypes of pEDV6-RFP- and pEDV6-TaCZSOD2-treated wheat plants inoculated with the virulent Pst race CYR31 at 12 dpi. (B) Quantification of H2O2 accumulation at 48 hpi in pEDV6-RFP- and pEDV6-TaCZSOD2-treated wheat plants inoculated with virulent Pst race CYR31. Values represent the means ± SE (n = 30). (C) Quantification of necrotic cell death area in TaCZSOD2-overexpression plants at 48 hpi. Values represent the means ± SE (n = 20). Differences between time-course points were assessed using Student’s t-tests. Asterisks indicate P < 0.05, double asterisks indicate P < 0.01. (D) H2O2 accumulation was observed by microscopy after DAB staining and necrotic cell death observed by epifluorescence. SV, substomatal vesicle. NC, necrotic cell death. Scale bars, 20 μm. (TIF) [file ppat.1010702.s015.tif]

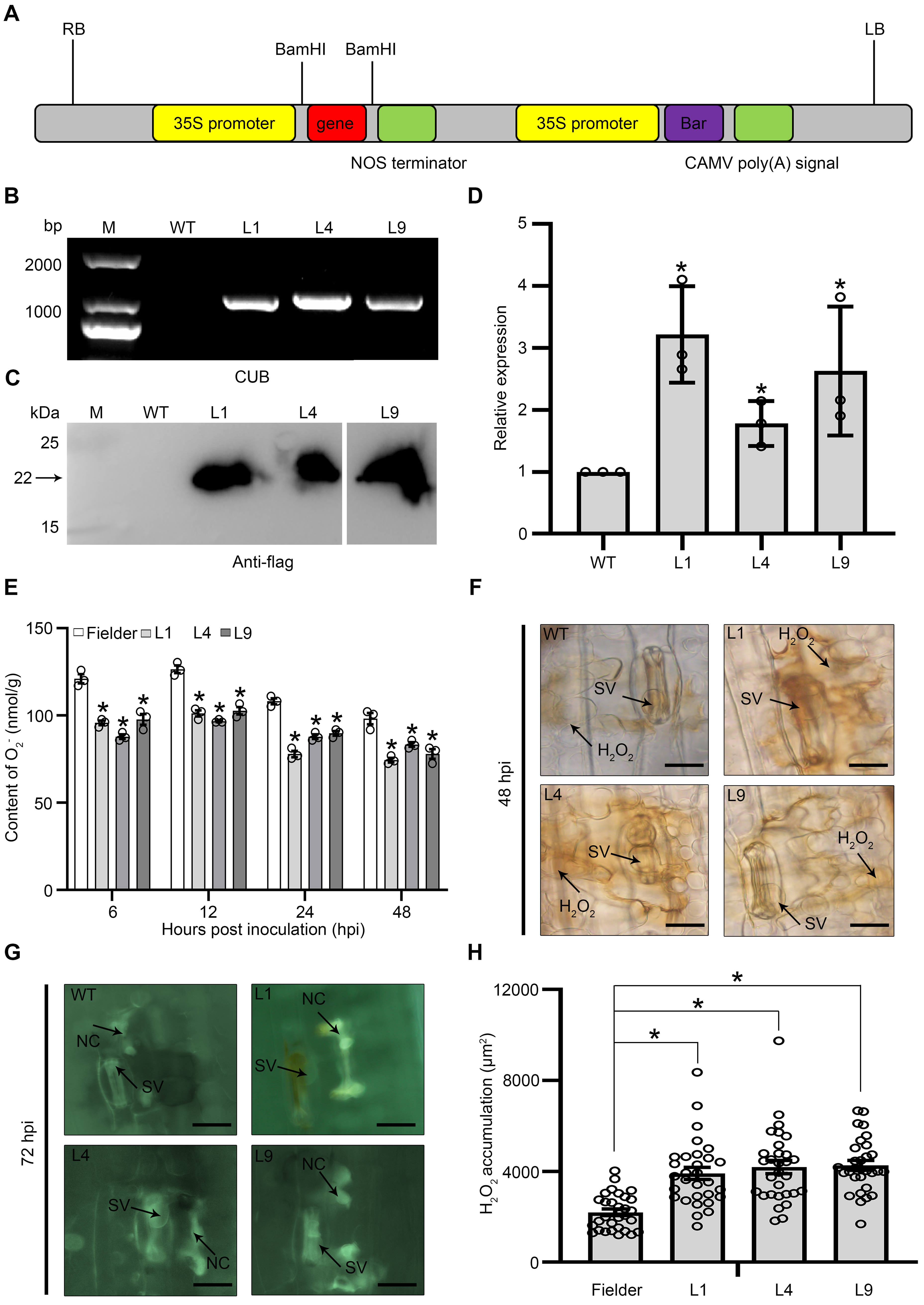

Supplement: S16 Fig — (A) Diagram showing the overexpression cassette in the wheat transformation construct CUB-TaCZSOD2-overexpression. LB, left border. RB, right border. (B-C) Transgenic plants were analyzed by genomic PCR and western blotting. (D) Relative transcript levels of TaCZSOD2 in TaCZSOD2-overexpression plants challenged by CYR31. TaEF-1α was used for normalization. Values represent the means ± SE (n = 3). (E) Content of O2− accumulation in different transgene lines at 6, 12, 24 and 48 hpi. Values represent the means ± SE of three independent samples. (F) H2O2 accumulation at infection sites were observed by microscopy after DAB staining. SV, substomatal vesicle. Scale bars, 20 μm. (G) Observation of necrotic cell death by epifluorescence in transgenic plants. NC, necrotic cell death. SV, substomatal vesicle. Scale bars, 20 μm. (H) Quantification of H2O2 accumulation in different transgenic lines at 48 hpi. Values represent the means ± SE (n = 30). Differences between time-course points were assessed using Student’s t-tests. Asterisks indicate P < 0.05. (TIF) [file ppat.1010702.s016.tif]

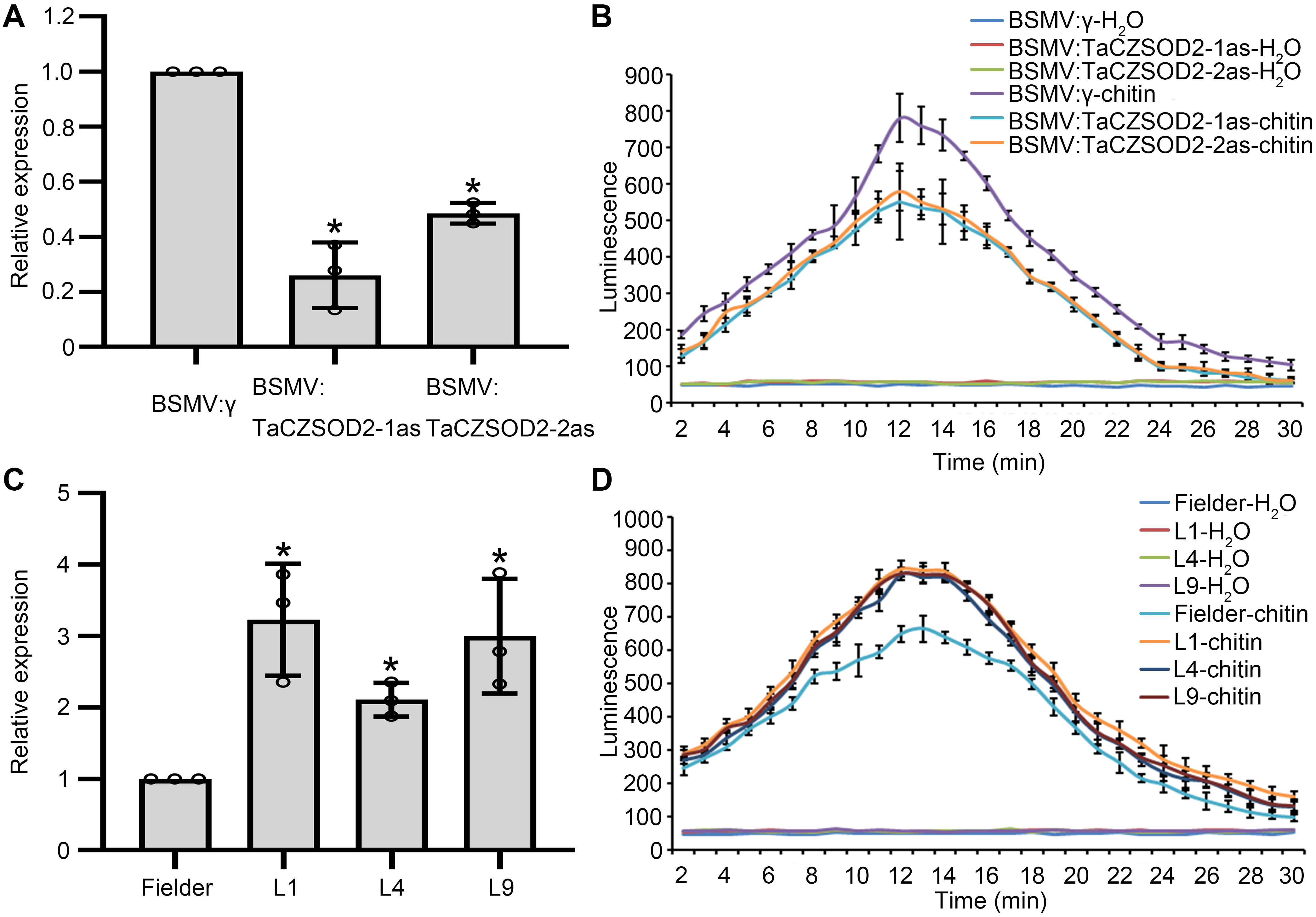

Supplement: S17 Fig — (A) Relative transcript levels of TaCZSOD2 in TaCZSOD2-knockdown plants were calculated by the comparative threshold (2-ΔΔCT) method. The quantitative qRT-PCR values were normalized to the expression level for TaEF-1α. Values represent the means ± SE (n = 3). (B) Reactive oxygen species (ROS) burst induced by 8 nM chitin in discs of TaCZSOD2-knockdown and WT leaves. Values represent the means ± SE (n = 6). (C) Relative transcript levels of TaCZSOD2 in TaCZSOD2-overexpression transgene lines were calculated by the comparative threshold (2-ΔΔCT) method. The quantitative qRT-PCR values were normalized to the expression level for TaEF-1α. Values represent the means ± SE (n = 3). (D) Reactive oxygen species (ROS) burst induced by 8 nM chitin in discs of TaCZSOD2-overexpression and WT leaves. Values represent the means ± SE (n = 6). (TIF) [file ppat.1010702.s017.tif]
