# Supplementary material for: Glycine-serine-rich effector PstGSRE4 in Puccinia striiformis f. sp. tritici inhibits the activity of copper zinc superoxide dismutase to modulate immunity in wheat
Source: PLoS Pathog. 2022 Jul 26;18(7):e1010702. doi: 10.1371/journal.ppat.1010702 (PMC9321418; doi:10.1371/journal.ppat.1010702)
Supplement: S1 File — (DOCX) [file ppat.1010702.s022.docx]

>TaSOD1.1_TraesCS2A02G121200.1

--------------------------------------------------------------------------------------------------------------------------------------------------QITHTMVKAVAVLTGSEGVKGT--------------------------IFFTQE-GEGPTTVTGSVTGLKEGLHGFHVHALGDT-------------TNGCMSTGPHFNPAGHVHGAPEDEIRHAGDLGNVTAGVDGV---ASINITDCHIPLTGP----------------------NSIVGRAVVVHGDA--DDLGKGG-------------HELSKSTGNAGARV--ACGII-GLQG--------------------------------------------------------------------------

>TaSOD1.2_TraesCS2A02G399000.1

MVGFLRAFTAASAVP-AAAVAAAALSSSSSSPSRSPSSRLRFPLP---PSLSAFAASSSSSAPVRAPTAAPPM-AAAATADLS-APDKGTA----------LPELTTEFMVDMKCEGCVTAVKNRLQTLEGIQNIEVDLNNQVVRVRGSLPVKIMLDALHQTGRDARLIGQGNPDDFLVSAAVAEFKGPVIFGVVRLAQV-NMELARVEATFSGLSPGKHGWSINEFGDL-------------TKGAESTGKVYNPQDYL------SDKPLGDLGTLEAGENGE---AQFSGSKEKLKV-------------------------VDLIGRSIALYATEDRSDPGIAA--------------AVVARSAGVGENYKKLCTCD-GVTIWESS----------------------------------------------------------------------

>TaSOD1.3_TraesCS2B02G417000.1

MVGFLRAFTAASAVP-AAAVAAVAL--SSSSPSRSPSSRLRFPLP---PSLSAFAASSASSSPVRAPTAAPPM-AAAATADLS-APDKGTA----------LPELTTEFMVDMKCEGCVTAVKNKLQTLEGIQNIEVDFNNQVVRVRGSLPVKLMLDALRQTGRDARLIGQGNPDDFLVSAAVAEFKGPVIFGVVRLAQV-NMELARVEATFSGLSPGKHGWSINEFGDL-------------TKGAESTGKVYNPQDYL------SDKLLGDLGTLEAGERE----AQFSGSKEKLKV-------------------------VDLIGRSIALYATEDRSDAGLAA--------------AVVARSAGVGENYKKLCTCD-GVTIWESS----------------------------------------------------------------------

>TaSOD1.5_TraesCS2D02G396500.1

MVGFLRAFTAASAVP-AAAVAAAAL-CSSSSPSRSPSSRLRFPLP----SLSAFAASSSSSSPVRAPTAAPPM-AAAATADLS-APDKGTA----------LPELTTEFMVDMKCEGCVTAVKNRLQTLEGIQNIEVDLNNQVVRVRGSLPVKIMLDALHQTGRDARLIGQGNPDDFLVSAAVAEFKGPVIFGVVRLAQV-NMELARIEATFSGLSPGKHGWSINEFGDL-------------TKGAESTGKVYNPQDYL------SDKPLGDLGTLEAGENGE---AQFSGSKEKLKV-------------------------VDLIGRSIALYATEDRSDPGIAA--------------AVVARSAGVGENYKKLCTCD-GVTIWESS----------------------------------------------------------------------

>TaSOD1.4_TraesCS2D02G123300.1

-------------------------------------------------------------------------------------------------------------------------------------------------------MVKAVAVLTGSEGVKGT--------------------------IFFTQE-GDGPTTVTGSVTGLKEGLHGFHVHALGDT-------------TNGCMSTGPHFNPAGHVHGAPEDEIRHAGDLGNVTAGADGV---ANINVTDCHIPLTGP----------------------NSIVGRAVVVHGDA--DDLGKGG-------------HELSKSTGNAGARV--ACGII-GLQG--------------------------------------------------------------------------

>TaSOD1.6_TraesCS4A02G065800.1

-------------------------------------------------------------------------------------------------------------------------------------------------MAGKPGSLKGVALISGGGADSAVA-----------------------GALHFVQDPSSGYTEVRGRVSGLAPGLHGFHIHAFGDT-------------TNGCNSTGPHFNPHNKSHGAPVDDERHVGDLGNIQANKDGV---AEIFIKDLQISLRGP----------------------HSILGRAVVVHADS--DDLGKGG-------------HELSKSTGNAGARI--GCGII-GIQPAV------------------------------------------------------------------------

>TaSOD1.7_TraesCS4B02G243200.1

-------------------------------------------------------------------------------------------------------------------------------------------------MAGKPGSLKGVALISGGSADSAVA-----------------------GALHFVEDPSSGYTEVRGRVSGLAPGLHGFHIHAFGDT-------------TNGCNSTGPHFNPHNKFHGAPMDDERHVGDLGNIQANKDGV---AEIFIKDLQISLRGP----------------------HSILGRAVVVHADS--DDLGKGG-------------HELSKSTGNAGARI--GCGVI-GIQPAV------------------------------------------------------------------------

>TaSOD1.8_TraesCS4D02G242800.1

-------------------------------------------------------------------------------------------------------------------------------------------------MAGKPVSLKGVALISGGAADSAVA-----------------------GALHFVQDPSSGYTEVRGWVSGLAPGLHGFHIHAFGDT-------------TNGCNSTGPHFNPHNKSHGAPVDDERHVGDLGNIQANKDGV---AEIFIKDLQISLRGP----------------------HSILGRAVVVHADS--DDLGKGP---------------------------------------WVTLDHD-------------------------------------------------------------------

>TaSOD1.9_TraesCS7A02G292100.1

----------------------MAA-----------------------QSLLFAAAAPLFQAPASARPFQS--------LRIVCTPEGATA--------------------------------------------------AARALVVADATKKAVAVLKGTSQVEGV--------------------------VTLTQE-DDGPTTVNVRITGLAPGLHGFHLHEFGDT-------------TNGCISTGPHFNPNGLTHGAPEDEVRHAGDLGNIVANAEGV---AETTIVDSQIPLTGP----------------------NAVVGRAFVVHELE--DDLGKGG-------------HELSLSTGNAGGRL--ACGVV-GLTPL-------------------------------------------------------------------------

>TaSOD1.10_TraesCS7B02G197300.1

----------------------MAA-----------------------QSLLFAAAAPLFQVPASARPFQS--------LRIVSTPGGATA--------------------------------------------------AARALVVADATKKAVAVLKGTSQVEGV--------------------------VTLTQE-DDGPTTVNVRITGLAPGLHGFHLHEFGDM-------------TNGCISTGPHFNPNGLTHGAPEDEVRHAGDLGNIVANAEGV---AETTIVDSQIPLTGP----------------------NAVVGRAFVVHELE--DDLGKGG-------------HELSLSTGNAGGRL--ACGVV-GLTPL-------------------------------------------------------------------------

>TaSOD1.11_TraesCS7D02G290700.1

----------------------MAA-----------------------QSLLFAAAAPLFQAPASARPFQS--------LRIVSTPGGATA--------------------------------------------------AARALVVADATKKAVAVLKGSSQVEGV--------------------------VTLTQE-DDGPTTVNVRITGLAPGLHGFHLHEFGDT-------------TNGCISTGPHFNPNGLTHGAPEDEVRHAGDLGNIVANAEGV---AETTIVDSQIPLTGP----------------------NAVVGRAFVVHELE--DDLGKGG-------------HELSLSTGNAGGRL--ACGVV-GLTPL-------------------------------------------------------------------------

>TaSOD2.1_TraesCS2A02G537100.1

----------------------MAL-----------------------RTLAAKKTLGLALGGARPLAAAR-----------------------------------------------------------------------GVATFTLPDLPYDFGALEPAVSGEIMR-------------------------LHHQKHHATYVANYNKALEQLDAAVSKGDASAVVHL--------QSAIKFNGGGHVNHSIFWKNLKPISEGGGEPPHGKLGWAIDEDFGSIEKLIKKMNAEGAALQGSGWVWLAL---------------DKEAKKLSVETTPNQ-DPLVTKGSNLYPLLGIDVWEHAYYLQYKNVRPDY--LTNIW-KVVNWKYAGEEYEKVLA-------------------------------------------------------------

>TaSOD2.2_TraesCS2B02G567600.1

----------------------MAL-----------------------RTLAAKKTLGLALGGAR-----------------------------------------------------------------------------GVATFTLPDLPYDYGALEPAVSGEIMR-------------------------LHHQKHHATYVANYNKALEQLDAAASKGDASAVVGL--------QSAIKFNGGGHVNHSIFWKNLKPISEGGGEAPHGKLGWAIDEDFGSIEKLIKKMNAEGAALQGSGWVWLAL---------------DKEAKRLSVETTPNQ-DPLVTKGSNLHPLLGIDVWEHAYYLQYKNVRPDY--LTNIW-KVVNWKYAGEEYEKVLA-------------------------------------------------------------

>TaSOD2.3_TraesCS2D02G538300.1

----------------------MAL-----------------------RTLAAKKTLGLALGGARPPAAAR-----------------------------------------------------------------------GVATFTLPDLPYDYGALEPAVSGEIMR-------------------------LHHQKHHATYVANYNKALEQLDAAVSKGDASAVVHL--------QSAIKFNGGGHVNHSIFWKNLKPISEGGGEAPHGKLGWAIDEDFGSIEKLIKKMNAEGAALQGSGWVWLAL---------------DKEAKRLSVETTPNQ-DPLVTKGSNLHPLLGIDVWEHAYYLQYKNVRPDY--LTNIW-KVVNWKYAGEEYEKVLA-------------------------------------------------------------

>TaSOD2.4_TraesCS4A02G390300.1

-----------------MLLPMRGL-----------------------PAAPPRPLAHPHASPAPPPPSLL--------SPRRRRPSRRLS--------------------------------------------------KVVSYYGLTTPPYKTDALEPYMSRRAVE-------------------------LHWGKHQQGHVDGLNKQL-AISP-LYGHTLEDLIKEAYNNGN--PLPEYNDAAEVWNHHFFWESMQ---PDGGGSPEAGVLQQIEKDFGSFFNFREEFMRSALSLLGSGWVWLVL---------------KRSERKLEVVHTRNAINPLAFGD---IPIISLDLWEHAYYLDYKDDRRTY--VSNFLDHLVSWHTVTLRMMRAEAFVNLGEPTIPVA-------------------------------------------------

>TaSOD2.5_TraesCS4A02G434000.1

-------MAFAAPVGVGGGPLSLAL-----------------------PASSSAPFLLRAGGDSPQRGRLRRLAAPRRGGARGDSRGRWNCHVTRCAGEANVVTEDDTANV-AADAAADQAANASGDAADV--SLNP-DDVDSVAWIKQQPLPYPADALEPYISKETVE-------------------------QHWGVHQRGHVDRLNGMIGGSE--WERMSIGQMMLASFNEGREPPHAPFFHAAQVWNHDFYWRSIK---PGGGGKPPERLLKFINRDFGSYDGMIKQFMDVALTQFGSGWVWLSYKGSKLPHVKSKSPIPSDNYGRLVISKSPNAINPLVWGH---SPLLAIDVWEHAYYLDYENRRAEY--VSAVLEKLVSWEMVESRLRKAVLRAIERDGHTSPKQRRKQLLSQAKSRVGDASTSGEARRRPRSKDQQAPSSVRMVPAGEAVPN

>TaSOD2.6_TraesCS7A02G048600.1

-------MVFAAPAGVGGRPLSLAL-----------------------PASSSAPFLLRDGGDSPQRGRLRRLTFPRRGGARGDSRGRWNCHITRCAGEANVVTEDDTANV-SADAAADKAADAIGDGADVLESLNP-DNADSVAWIKQQPLPYPSDALEPYISKETVA-------------------------QHWGVHQHMHVDRLNGMIGGSE--WERMSIGQMMLASFNEGREPPHAPFFHAAQVWNHDFYWRSMK---PGGGGKPPERLLKFINRDFGSYDGMIKQFMDAALTQFGSGWVWLSYKGSKLPHVKSKSPIPSDNYGRLVISKSPNAINPLVWGH---SPLLAIDVWEHAYYLDYENRRAEY--VSAVLEKLVSWEMVESRLRKAVLRAIERDGHTSPKQRRKQLLSQARSRVGDASTSGEARRRPRSKDQQAPSSVTMVPAGEAVPN

>TaSOD2.7_TraesCS7A02G090400.1

-----------------MLLPMRGL-----------------------PAAPPRPLAHPHTAPAPPPSLLG---------PRRRRPSRRLS--------------------------------------------------KVVSYYGLTTPPYKTDALEPYMSRRAVE-------------------------LHWGKHQQDYVDGLNKQL-AISP-LYGHTLEDLIKEAYNNGN--PLPEYNDAAEVWNHHFFWESMQ---PEGGGSPEAGVLQQIEKDFGSFFNFREEFMRSALSLLGSGWVWLVL---------------KRSERKLEVVHTRNAINPLAFGD---IPIISLDLWEHAYYLDYKDDRRTY--VSNFLDHLVSWHTVTLRMMRAEAFVNLGEPTIPVA-------------------------------------------------

>TaSOD2.8_TraesCS7D02G043000.1

-------MVFAAPAGVGGGPLSVAP-----------------------PASSSAPFLLRAGGDSPQRGMLRRLTFPRRGGARGQSQRRWDCHITRCAGKANVVTEDDTAKVAAADATADQAAD---DTADVLESLNPDDDVDSVAWIKQQPLPYPADALEPYISKETVE-------------------------QHWGVHQRMHVDRLNGMIGGSE--WERMSIGQMMLASFNEGREPPHAPFFHAAQVWNHDFYWRSMK---PGGGGKPPERLLKFINRDFGSYDGMIKQFMDAALTQFGSGWVWLSYKGSKLPHVKSKSPIPSDNYGRLVISKSPNAINPLVWGH---SPLLAIDVWEHAYYLDYENRRAEY--VSAVLEKLVSWEMVESRLRKAVLRAIERDGHTSPKQRRKQLVSQAKGRVGDASTSGEARRRPRSKDQQAPSSVTMVPAGEAVPN

>TaSOD2.9_TraesCS7D02G086400.1

-----------------MLLPVRGL-----------------------PAAPHRPLTHPHSSPAPPPSLLG---------ARRRRPSRRLS--------------------------------------------------KVVSYYGLTTPPYKTDALEPYMSRRAVE-------------------------LHWGKHQQEYVDGLNRQL-AISP-LYGHTLEDLIKEAYNNGN--PLPEYNDAAEVWNHHFFWESMQ---PEGGGSPEAGVLQQIEKDFGSFFNFREEFMRSALSLLGSGWVWLVL---------------KRSERKLEVVHTRNAINPLAFGD---IPIISLDLWEHAYYLDYKDDRRTY--VSNFLDHLVSWHTVTVRMMRAEAFVNLGEPTIPVA-------------------------------------------------

>AtCSD1_AT1G08830.1

-------------------------------------------------------------------------------------------------------------------------------------------------------MAKGVAVLNSSEGVTGT--------------------------IFFTQE-GDGVTTVSGTVSGLKPGLHGFHVHALGDT-------------TNGCMSTGPHFNPDGKTHGAPEDANRHAGDLGNITVGDDGT---ATFTITDCQIPLTGP----------------------NSIVGRAVVVHADP--DDLGKGG-------------HELSLATGNAGGRV--ACGII-GLQG--------------------------------------------------------------------------

>AtCSD2_AT2G28190.1

----------------MAATNTILA-----------------------FSSPSRLLIPPSSNPSTLRSSFRGVSLNNNNLHRLQSVSFAVK-------------------------------------------------APSKALTVVSAAKKAVAVLKGTSDVEGV--------------------------VTLTQD-DSGPTTVNVRITGLTPGPHGFHLHEFGDT-------------TNGCISTGPHFNPNNMTHGAPEDECRHAGDLGNINANADGV---AETTIVDNQIPLTGP----------------------NSVVGRAFVVHELK--DDLGKGG-------------HELSLTTGNAGGRL--ACGVI-GLTPL-------------------------------------------------------------------------

>AtMSD1_AT3G10920.1

----------------------MAI-----------------------RCVASRKTLAGLKETSSRLLRIR-----------------------------------------------------------------------GIQTFTLPDLPYDYGALEPAISGEIMQ-------------------------IHHQKHHQAYVTNYNNALEQLDQAVNKGDASTVVKL--------QSAIKFNGGGHVNHSIFWKNLAPSSEGGGEPPKGSLGSAIDAHFGSLEGLVKKMSAEGAAVQGSGW-WLGL---------------DKELKKLVVDTTANQ-DPLVTKGGSLVPLVGIDVWEHAYYLQYKNVRPEY--LKNVW-KVINWKYASEVYEKENN-------------------------------------------------------------

>AtMSD2_AT3G56350.1

-----------------MTTTVIII-----------------------IFVAIFATTLHDARGATMEPCLE-----------------------------------------------------------------------SMKTASLPDLPYAYDALEPAISEEIMR-------------------------LHHQKHHQTYVTQYNKALNSLRSAMADGDHSSVVKL--------QSLIKFNGGGHVNHAIFWKNLAPVHEGGGKPPHDPLASAIDAHFGSLEGLIQKMNAEGAAVQGSGWVWFGL---------------DRELKRLVVETTANQ-DPLVTKGSHLVPLIGIDVWEHAYYPQYKNARAEY--LKNIW-TVINWKYAADVFEKHTRDLDIN--------------------------------------------------------

>AtFSD1_AT4G25100.1

------------------------------------------------MAASSA----------------------------------------------------------------------------------------VTANYVLKPPPFALDALEPHMSKQTLE-------------------------FHWGKHHRAYVDNLKKQVLGTE--LEGKPLEHIIHSTYNNGD--LLPAFNNAAQAWNHEFFWESMK---PGGGGKPSGELLALLERDFTSYEKFYEEFNAAAATQFGAGWAWLAY-----------------SNEKLKVVKTPNAVNPLVLGS---FPLLTIDVWEHAYYLDFQNRRPDY--IKTFMTNLVSWEAVSARLEAAKAASA----------------------------------------------------------

>AtCSD3_AT5G18100.1

-------------------------------------------------------------------------------------------------------------------------------------------------MEAPRGNLRAVALIAGDNNVRGC--------------------------LQFVQD-ISGTTHVTGKISGLSPGFHGFHIHSFGDT-------------TNGCISTGPHFNPLNRVHGPPNEEERHAGDLGNILAGSNGV---AEILIKDKHIPLSGQ----------------------YSILGRAVVVHADP--DDLGKGG-------------HKLSKSTGNAGSRV--GCGII-GLQSSADAKL--------------------------------------------------------------------

>AtFSD3_AT5G23310.1

--------------------MSSCV-----------------------VTTSCFYTISDSSIRLKSPKLLN----LSNQQRRRSLRSRGGL--------------------------------------------------KVEAYYGLKTPPYPLDALEPYMSRRTLE-------------------------VHWGKHHRGYVDNLNKQL-GKDDRLYGYTMEELIKATYNNGN--PLPEFNNAAQVYNHDFFWESMQ---PGGGDTPQKGVLEQIDKDFGSFTNFREKFTNAALTQFGSGWVWLVL---------------KREERRLEVVKTSNAINPLVWDD---IPIICVDVWEHSYYLDYKNDRAKY--INTFLNHLVSWNAAMSRMARAEAFVNLGEPNIPIA-------------------------------------------------

>AtFSD2_AT5G51100.1

MMNV---------------AVTATP-----------------------SSLLYSPLLLPSQGPNRRMQWKR--------NGKRRLGTKVAV------------------------------------------------SGVITAGFELKPPPYPLDALEPHMSRETLD-------------------------YHWGKHHKTYVENLNKQILGTD--LDALSLEEVVLLSYNKGN--MLPAFNNAAQAWNHEFFWESIQ---PGGGGKPTGELLRLIERDFGSFEEFLERFKSAAASNFGSGWTWLAYKANRLDVANAVNPLPKEEDKKLVIVKTPNAVNPLVWDY---SPLLTIDTWEHAYYLDFENRRAEY--INTFMEKLVSWETVSTRLESAIARAVQREQEGTETEDEENPDDEVPEVYLDSDIDVSEVD------------------------

>OsSOD2_LOC-Os03g11960

-------------------------------------------------------------------------------------------------------------------------------------------------MAGKAGGLKGVALIGGAGGNSAVA-----------------------GALHFFQDPSTGYTEVRGRVTGLAPGLHGFHIHSFGDT-------------TNGCNSTGPHFNPHNKSHGAPSDDERHVGDLGNIVANKDGV---ADIFIKDLQISLSGP----------------------HSILGRAVVVHADS--DDLGRGG-------------HELSKTTGNAGTRI--GCGII-GLRSAV------------------------------------------------------------------------

>OsSOD1_LOC-Os03g22810

-----------------MVQVISDE-----------------------LRLSPLTGRALNTGQRRIYPVVPNYLDTGSSAKELGVTERRQS-------------------VTLGARALNVGWWLQLQAASLTTNTTAHRWREGNLPEAPDSKPAGVASPPPSSSSSSPRGSPEITLTMVKAVVVLGSSEIVKGTIHFVQE-GDGPTTVTGSVSGLKPGLHGFHIHALGDT-------------TNGCMSTGPHYNPAGKEHGAPEDETRHAGDLGNVTAGEDGLFFFPLFRIFICSIPV-----------------------------------------NDLT--------------------------------------------------------------------------------------------------------------------

>OsSOD-Cu/Zn_LOC-Os04g48410

MVGFLRALTAASAVPAAAAVAAVAL-----STNSSSSSRLRLPSPASLPSLSSAYAAAPASGSARKPNAVPPMAAAAATADLSAAADKGAA----------LPELMTEFMVDMKCDGCVTAVKNKFQTLEGIKNIEVDLNNQVVRVLGSLPVNTMLDTLHQTGRDARLIGQGNPNDFLVSAAVAEFKGPVIFGVVRLAQV-NMELAIVEATFSGLSPGKHGWSINEFGDL-------------TRGAESTGKVYNPSDYR------SNKPLGDLGTLEAGEKGE---AQFSASKEKLKV-------------------------VDLIGRSIALYATEDRSDPGIAA--------------AVIARSAGVGENYKKLCTCD-GVTIWESS----------------------------------------------------------------------

>OsSODA1_LOC-Os05g25850

----------------------MAL-----------------------RTLASRKTLAAAALPLAAAAAAR-----------------------------------------------------------------------GVTTVALPDLPYDYGALEPAISGEIMR-------------------------LHHQKHHATYVANYNKALEQLDAAVAKGDAPAIVHL--------QSAIKFNGGGHVNHSIFWNNLKPISEGGGDPPHAKLGWAIDEDFGSFEALVKKMSAEGAALQGSGWVWLAL---------------DKEAKKLSVETTANQ-DPLVTKGANLVPLLGIDVWEHAYYLQYKNVRPDY--LSNIW-KVMNWKYAGEVYENATA-------------------------------------------------------------

>OsFSD1.1_LOC-Os06g02500

--MAFATLVGVGGLSPALFSPSRPLSCSSST-----------------SVSAPFILRAGGGGDARRHGLRRLVTPLRGSACRGESTNSRVL----QCANEANVVTEDDIVNDGIDDETASDAEMDEDAEANGDESSGTDEDASVSWIEQQPLPYPSDALEPYISKETVE-------------------------QHWGVHQNIHVERLNGMIGGSE--WEGMSLGQMMLSSFNEGREAPHPPFFHAAQIWNHDFYWRSMQ---PGGGGKPPERLLKFINRDFGSYDGMIRQFMDAASTQFGSGWVWLCYKTSKLPHVKSRSPIPSDNYGRLVISKSPNAINPLVWGH---SPLLAIDLWEHAYYLDYEDRRSDY--VSTFLEKLVSWETVESRLKKAVQRAVERDEYVSTKHIRKQLLARAKSQIRAMPQQVNGDAREQTSGQEKSLGV-----------

>OsFSD1.2_LOC-Os06g05110

------------------------------------------------MAAFASALRVLPSPPAAVPRRLR------SREQRQGCRSRRYS--------------------------------------------------KVVAYYALTTPPYKLDALEPYISKRTVE-------------------------LHWGKHQQDYVDSLNKQLATSM--FYGYTLEELIKEAYNNGN--PLPEYNNAAQVWNHHFFWESMQ---PEGGGSPGRGVLQQIEKDFGSFTNFREEFIRSALSLLGSGWVWLVL---------------KRKERKFSVVHTQNAISPLALGD---IPLINLDLWEHAYYLDYKDDRRMY--VTNFIDHLVSWDTVTLRMMRAEAFVNLGEPNIPVA-------------------------------------------------

>OsCSD1_LOC-Os07g46990

-------------------------------------------------------------------------------------------------------------------------------------------------------MVKAVAVLASSEGVKGT--------------------------IFFSQE-GDGPTSVTGSVSGLKPGLHGFHVHALGDT-------------TNGCMSTGPHFNPTGKEHGAPQDENRHAGDLGNITAGADGV---ANVNVSDSQIPLTGA----------------------HSIIGRAVVVHADP--DDLGKGG-------------HELSKTTGNAGGRV--ACGII-GLQG--------------------------------------------------------------------------

>OsCSD2_LOC-Os08g44770

-----------MQAILAAAMAAQTL-----------------------LFSATAPPASLFQSPSSARPFHS--------LRLAAGPAGAAA---------------------------------------------------ARALVVADATKKAVAVLKGTSQVEGV--------------------------VTLTQD-DQGPTTVNVRVTGLTPGLHGFHLHEFGDT-------------TNGCISTGPHFNPNNLTHGAPEDEVRHAGDLGNIVANAEGV---AEATIVDKQIPLSGP----------------------NSVVGRAFVVHELE--DDLGKGG-------------HELSLSTGNAGGRL--ACGVV-GLTPL-------------------------------------------------------------------------
