# Supplementary material for: Glycine-serine-rich effector PstGSRE4 in Puccinia striiformis f. sp. tritici inhibits the activity of copper zinc superoxide dismutase to modulate immunity in wheat
Source: PLoS Pathog. 2022 Jul 26;18(7):e1010702. doi: 10.1371/journal.ppat.1010702 (PMC9321418; doi:10.1371/journal.ppat.1010702)
Supplement: Attachment — Submitted filename: Response to comments on PstGSRE4 ms.docx [file ppat.1010702.s024.docx]

Re: PPATHOGENS-D-22-00159

Glycine-serine-rich effector PstGSRE4 in *Puccinia striiformis* f. sp. *tritici* inhibits the activity of copper zinc superoxide dismutase to modulate immunity in wheat

Dear Editors,

Thank you very much for handling our manuscript. We are grateful to the reviewers for their valuable comments, which certainly contribute to a significant improvement of the manuscript. In the revised manuscript, we have attempted to address all the comments from you and the reviewers. Below are our point-to-point responses to the comments from the reviewers. Thank you for your favorable consideration.

Best regards,

Jun Guo and Zhensheng Kang

College of Plant Protection

Northwest A&F University

Yangling, Shaanxi, 712100

PR China

Phone/Fax: +86 29 87082439

Email: guojunwgq@nwsuaf.edu.cn

**Reviewer 1 Comments for the Author**

The work presented by Liu et al shows that a new glycine-serine-rich effector, PstGSRE4, reduces H_2_O_2_ accumulation and HR areas to facilitate *Puccinia striiforms* f. sp. *tritici* (*Pst*) infection, and is highly induced during the early stages of infection. Moreover, PstGSRE4 inhibits the enzyme activity of wheat copper zinc superoxide dismutase TaCZSOD2, which acts as a positive regulator of wheat resistance to *Pst*. Such systematic research results provide new insights into the molecular mechanisms of GSREs of rust fungi in regulating plant immunity. Overall, I think this research is a major advancement in the plant-microbe interaction field, especially the pathogenic mechanism of biotrophic fungi.

Here I list several points or suggestions that I believe would improve the manuscript.

**Minor Issues:**

1. In the manuscript, the author described that PstGSRE4 inhibits the enzyme activity of TaCZSOD2 to facilitate *Pst* infection, but the part of “TaCZSOD2 positively regulates wheat resistance against *Pst*”, showed that transcript levels of TaCZSOD2 were up-regulated at 12, 48, and 96 hpi with the avirulent *Pst* race CYR23, and up-regulated at 96 and 120 hpi with the virulent *Pst* race CYR31. Here, I have several questions. First, does the transcript levels increasing represent the increasing the protein level?

**Response:** Thanks for your advice. Due to unavailability of antibody of TaCZSOD2, we cannot detect the protein level of TaCZSOD2 during incompatible and compatible interaction between wheat and *Pst*. Therefore, it is unavailable for us to make sure the transcript levels of *TaCZSOD2* increasing represent the increasing of the protein level of TaCZSOD2. To investigate the correlation of the transcript level and the protein level of TaCZSOD2, we detect the transcript level and protein level of *TaCZSOD2* in *TaCZSOD2*-overexpression (OE) T_2_ transgenic lines. The results indicated that both the transcript level and protein level of TaCZSOD2 are increased in *TaCZSOD2*-OE T_2_ transgenic lines compared with the Fielder (control) plants (**new S16C and S16D Fig**), suggesting that the transcript levels of *TaCZSOD2* may correlate with its protein level.

Second, CYR23 is an avirulent strain, while CYR31 is a virulent strain, the authors need to illustrate why these two strains cause different expression profiles of TaCZSOD2.

**Response:** In a previous study, wang et al. (2007) use 3,3-diamino-benzidine (DAB) to analyze the generation of H_2_O_2_ and found that in the incompatible interaction during 12-24 hours post-inoculation (hpi), the percentage of penetration sites with DAB staining increased gradually with time. With extended incubation period, percentage of infection sites with DAB staining decreased until 48 hpi with a slightly higher percentage at 36 hpi, and increased again at 60 hpi. In the compatible interaction, only a low frequency of infection sites exhibiting DAB staining was observed at 12 hpi. During the further incubation period until 72 hpi, a very small portion of infection sites showed DAB staining at low intensity. However, at 96 hpi, the percentage of penetration sites with DAB staining increased rapidly even to a higher level than in incompatible interaction. In this study, our results also indicated that transcript levels of *TaCZSOD2* were up-regulated at 12, 48 and 96 hpi with the avirulent *Pst* race CYR23 (incompatible interaction), and up-regulated at 96 and 120 hpi with the virulent *Pst* race CYR31 (compatible interaction) (**new** **S14B Fig**). Meanwhile, H_2_O_2_ accumulation triggered by *Pst* race CYR31 was significantly increased in *TaCZSOD2*-overexpression plants at 6, 12, 24 and 48 hpi (**new** **Fig 5D**). In higher plants, CuZnSOD is the main antioxidant defense enzyme, it catalyzes superoxide anion (O_2_^−^) radical disproportionation to produce O_2_ and H_2_O_2_ (Dreyer et al. 2019). All the data suggest that the different transcript profiles of *TaCZSOD2* during incompatible and compatible interaction is dependent on the function of TaCZSOD2 in wheat challenged by *Pst*.

Reference:

1. Wang CF, Huang LL, Buchenauer H, Han QM, Zhang HC, Kang ZS. Histochemical studies on the accumulation of reactive oxygen species (O_2_^-^ and H_2_O_2_) in the incompatible and compatible interaction of wheat-*Puccinia striiformis* f. sp. *tritici.* Physiol Mol Plant Pathol. 2007;71: 230-239. doi: 10.1016/j.pmpp.2008.02.006
2. Dreyer BH, Schippers JHM. Copper-zinc superoxide dismutases in plants: evolution, enzymatic properties, and beyond. Ann Plant Rev. 2019;2: 1-36. doi: [10.1002/9781119312994.apr0705](https://doi.org/10.1002/9781119312994.apr0705)

Third, which strain includes the effector PstGSRE4? In the model, I can see PstGSRE4 is from the CYR31 strain, but the part of “Relative transcript levels of PstGSRE4 at different *Pst* infection stages”, showed that PstGSRE4 is from CYR32.

**Response:** In our study, BLASTp analyses revealed that homologs of PstGSRE4 can be found only in rust fungi (**S1 and S2 Table**), suggesting that PstGSRE4 is conserved in rust fungi. To investigate whether PstGSRE4 is conserved in CYR31, CYR32 and CYR23, we cloned the *PstGSRE4* genes using complementary DNA from *Pst* CYR31, CYR32 and CYR23. Sequence analysis indicated that PstGSRE4 is highly conserved (97.7% identity) among CYR31, CYR32 and CYR23 and the result is shown as follow.

2. The author should add some experiments to prove that PstGSRE4 depends on which pathway to suppresses Pst322- and Bax-induced cell death.

**Response:** According to your advice, we performed the DAB staining experiment to detect the H_2_O_2_ production in *N. benthamiana*. Our results indicated that the DAB staining in the leaf regions infiltrated with Bax or Pst322 accumulated a large number of H_2_O_2_ compared with those infiltrated with MgCl_2_ buffer (**S5A and S5B Fig**), suggesting that Bax- or Pst322-trigged cell death may be due to the accumulation of H_2_O_2_. And the DAB staining in the leaf regions infiltrated with PstGSRE4/Bax or PstGSRE4/Pst322 was much weaker compared with those infiltrated with buffer/Bax or buffer/Pst322 (**S5C and S5D Fig**). These results suggested that PstGSRE4 suppresses Bax- and Pst322-triggered cell death by inhibiting the accumulation of H_2_O_2_ triggered by Bax and Pst322. H_2_O_2_ is considered to be important ROS in response to biotic stress (wang et al. 2007). All the data suggest that PstGSRE4 may suppress Pst322- and Bax-induced cell death depending on ROS pathway.

Reference:

Wang CF, Huang LL, Buchenauer H, Han QM, Zhang HC, Kang ZS. Histochemical studies on the accumulation of reactive oxygen species (O_2_^-^ and H_2_O_2_) in the incompatible and compatible interaction of wheat-*Puccinia striiformis* f. sp. *tritici.* Physiol Mol Plant Pathol. 2007;71: 230-239. doi: 10.1016/j.pmpp.2008.02.006

3. ROS burst as an important signal after pathogen infection, and the manuscript proved that TaCZSOD2 is a positive regulator of wheat immunity. I would suggest to identify the ROS burst level in the knockdown and overexpression lines of TaCZSOD2 treated with flg22 or Chitin.

**Response:** Thank you for your suggestions. We have performed the experiments and the results are shown in **new S17 Fig**.

4. Whether GSRE1 interact with CZSOD2 in the Y2H system?

**Response:** We performed the Y2H experiments and found that PstGSRE1 cannot interact with TaCZSOD2. The result is shown in **new S1C Fig**.

5. Response to comment: Please correct the spelling mistakes in lines 178, 518, 586, 653, et al.

**Response:** Revised.

6. Lines 254 and 255, “The hyphal areas during infection of L19 and L76 were significantly reduced at 48 and…..”, I think “significantly reduced” is not an acceptable description based on the result showed in Fig. 1D.

**Response:** Thanks for your advice and we revised the sentence as “The results showed that hyphal areas during infection of L19 and L76 significantly reduced at 120 hpi”.

7. The description is not clear in lines 445-447.

**Response:** We have rephrased the description as “Overexpression of *ΔTP-TaCZSOD2* in *N. benthamiana* improved the CuZnSOD enzyme activity, but slightly lower than overexpression of full length of *TaCZSOD2*, suggesting that TaCZSOD2 of the chloroplast can also be activated via the CCS-independent pathway when localized in cytoplasm like CSD2 in *Arabidopsis*” in the revised manuscript.

8. “d” or “day”, for example in line 564 and line 571, they should be uniform.

**Response:** Revised.

**Reviewer 2 Comments for the Author**

The paper describes the function of a glycine-serine-rich effector protein from *Puccinia striiformis* f. sp. *tritici* (*Pst*). The authors demonstrated that this protein inhibits the activity of one of the wheat copper zinc superoxide dismutases (TaCZSOD2) and this way prevents H_2_O_2_ accumulation and promotes virulence. The results are well described and the conclusions taken are correct. The results provide a mechanistic understanding on how fungal pathogens manipulate plant immunity. Although this has been addressed in many works, it still remains to be unveiled. My main concern is related to the information provided in the materials and methods. Sometimes I found this information scarce or not clear enough.

**Major Issues:**

1. In the introduction, it will be important to include information about the differences in activity between O_2_^-^ and H_2_O_2_ since this is critical to understand the results of the work.

**Response:** We have rephrased the introduction section according to your suggestion as “ROS burst is generally defined as a rapid production of high levels of ROS in response to external stimuli [5]. Superoxide radicals (O_2_^-^) and hydrogen peroxide (H_2_O_2_) are considered important ROS in response to biotic stress [6]. During plant-pathogen interaction, penetration of pathogen into host plasma membrane triggers the early O_2_^−^ burst by an NADPH oxidase, then they are rapidly converted to H_2_O_2_ by dismutation [5]. Most of the data seem to indicate that the major ROS building the oxidative burst is H_2_O_2_, with possible participation of O_2_^−^”.

2. My main concern is related to the description on how the activity assays of CuZnSOD were performed. Was the protein purified? If so, the methodology should be described. How was the NBT reaction performed? How was the concentration of the proteins determined?

**Response:** The activity assays of CuZnSOD were performed with purified proteins. We added more description about protein purification, performance of NBT reaction, and proteins concentration determination in Materials and methods section.

3. In the infection assays, line 639: How was the inoculum obtained?

**Response:** We described it in our manuscript as “Freshly collected urediospores of *Pst* race CYR23 were obtained from the leaves of wheat cultivar Thatcher (MX169), while CYR31 and CYR32 were obtained from Suwon11”.

4. Determination of accumulation of O_2_^-^ and H_2_O_2_. How was it performed? what is the kit consisting in?

**Response:** We have revised and included all the information in Materials and methods section.

5. For most of the experiments, what is considered a biological replicate? Were the experiments independent?

**Response:** “Three biological replicates” means the experiments were independently performed three times, with each biological replicate contains three technical repeats.

6. I could not find information about how the qPCR was performed.

**Response:** We have rephrased this part and please see qRT-PCR analysis in Materials and methods section.

7. Why the estimation of H_2_O_2_ content was performed using different methodologies in fig1 and 2? Both experiments should use the same methodology.

**Response:** Thanks for your advice. We have performed the experiments and the results shown in **new Fig 1D, Fig 5D, S9E Fig and S16E Fig**.

8. The legend of figure 6 is not complete. Specially 6D and 6F need more information about the experiment.

**Response:** We have rephrased the **legend of new Fig 6** and Materials and methods section.

**Minor Issues:**

1. A large section of the abstract contains information about a previosly identified effector protein. I would recommend reduce this part to a minimum.

**Response:** Thanks for your advice. We have rephrased this part as “In a previous study, we functionally characterized a glycine-serine-rich effector PstGSRE1 with a glycine-serine-rich motif (m9)”.

2. Indicate what is the meaning of TP.

**Response:** TP means chloroplast transit peptide and we also add it in the manuscript.

3. Material and methods. There are several references of the products that are missing: Line 609, Line 613.

**Response:** Revised.

4. Lines 490-493. What do the authors mean with "at certain levels"? Could this sentence be more precise?

**Response:** We have revised the sentence as “Thus, our results proved that PstGSRE4 inhibits the activity of total CuZnSOD by inhibiting the enzyme activity of TaCZSOD2 to control moderate H_2_O_2_ accumulation upon *Pst* infection, and further promote stripe rust disease”.

5. In figure 1D, hyphal areas are quantified. What is this exactly and how was it performed?

**Response:** We have rephrased this part in Materials and methods section.

**Reviewer 3 Comments for the Author**

Liu et al. present work on the molecular characterization of the effector PstGSRE4 and its host target TaCuZnSOD2. Based on previous work on the effector PstGSRE1, they identified members of the glycine/serine-rich effector family (GSRE). PstGSRE4 was found to lack the m9 domain previously found in PstGSRE1 and yeast two-hybrid experiments found that PstGSRE4 did not interact with TaLOL2, the target of PstGSRE1. Secretion assays in yeast found that the signal peptide of PstGSRE4 is functional. PstGSRE4 is expressed early in the interaction with wheat and most highly expressed between 24-48 hpi. They found that infiltration of Pseudomonas fluorescence carrying PstGSRE4 reduced callose formation, indicating that PstGSRE4 can contribute to the suppression of PTI responses. Similar results were observed for transient expression in wheat using Agrobacterium-mediated transformation. Host induced gene silencing of PstGSRE4 using two distinct fragments were integrated into BSMV and caused a reduction in PstGSRE4 transcript accumulation and led to reduced *Pst* biomass. Transgenic wheat lines expressing an RNAi construct targeting PstGSRE4. Similar to HIGS experiments with BSMV, the stable RNAi caused a reduction in *Pst* development and an increase in ROS formation (H_2_O_2_). Transgenic wheat lines carrying overexpression constructs of PstGSRE4 led to a decrease in ROS formation and increase in Pst development. A yeast two-hybrid screen identified TaCuZnSOD2 as a putative interactor. The interaction was confirmed with expression in E. coli based on pulldown of recombinant PstGSRE4 and TaCuZnSOD2 and Co-IP in Nicotiana benthamiana. Fluorescently tagged PstGSRE4 and TaCuZnSOD2 were found to colocalise in the cytoplasm. Transient silencing of TaCuZnSOD2 using BSMV found that it positively regulates immunity to Pst to an avirulent isolate (CYR23) but not a virulent isolate (CYR31). Enzymatic activity was also reduced. Expression of recombinant proteins of PstGSRE4 and TaCuZnSOD2 showed that PstGSRE4 inhibits the activity of TaCuZnSOD2 in vitro and in vivo. Collectively, this is a systematic study that used almost every available molecular technique to investigate the molecular function of PstGSRE4 and its target, TaCuZnSOD2.

**Major Issues:**

No single major issue was identified, but considerable minor issues were found that collectively become a major issue, particularly in data presentation.

**Response:** Thanks for your advices.

**Minor Issues:**

1. For all bar plots in the manuscript, raw data points should be included within each figure. This is even more important when only three biological replicates were performed. Also include the number of fields that were analysed for all the microscopy experiments.

**Response:** According to your advice, we have revised all Figures.

2. Change transient for stable transformation.

**Response:** Revised.

3. Figure 3A. Include a negative control for TaCZSOD2 in the yeast-two-hybrid experiment.

**Response:** According to your advice, we performed the Y2H experiment and the results are shown in **new Fig 3A**.

4. Figure 3B. There doesn’t seem to be enrichment of PstGSRE4-GST in the GST pulldown experiment with TaCZSOD1-His. Do you have any idea of why this might have happened? The pulldown should be enriching the GST-tagged effector.

**Response:** Thanks for your advice. We have repeated this experiment and the results are shown in **new Fig 3B**.

5. Figure 3C. PstGSRE4-GFP is not present in the input of the CoIP experiments, yet it is detected in other experiments (e.g. Fig. S4C, Fig. S12A). Can you explain why this might be?

**Response:** Thanks for your advice. We checked our original data and made a **new Fig 3C** with better result. However, the results showed that the band of PstGSRE4-GFP of **new Fig 3C** was still weaker than that of **new Fig S13A**, suggesting that co-expression of two proteins might influence the amount of each protein expression.

6. Figure 4C. "BSMV:" should be removed. The expression of the other TaCZSODs was checked, but these were not targeted with BSMV.

**Response:** Revised.

7. S1 Fig. State which of the four proteins in panel A are GSRE1 and GSRE4. State the number of replicate experiments performed for panel C.

**Response:** We have added it in **legend of new S1 Fig**.

8. S4 Fig and S12 Fig. Within the figure legend, list the predicted protein sizes for all five recombinant proteins shown in panel C.

**Response:** We have added it in **legend of S4 and new S13 Fig**.

9. The legend title for S5 Fig is incorrect, change ‘S’ to 5. Which wheat accession was used in this experiment?

**Response:** We have corrected the legend title and added the wheat accession in **legend of new S6A Fig**.

10. S6 Fig. State what is shown in Panel A. Is this genomic DNA or cDNA?

**Response:** Revised.

11. S7 Fig. How many sites were evaluated for Panel B/C? State in legend.

**Response:** Thanks for your advice. Thirty sites for Panel B and twenty sites for Panel C. We also revised in legend.

12. S8 Fig. B. Marker instead of Maker.

**Response:** Revised.

13. For length of scale bar, state “left” and “right” for 20 and 50 um, respectively.

**Response:** Revised.

14. S10 Fig. Alignment of the proteins needs to publicly deposited (Figshare) or provided as supplemental data. The number of bootstraps performed should be included the legend. The methods for tree construction were not present in the Materials and Methods. State that this is an unrooted tree. State explicitly in the legend that this is based on a protein alignment.

**Response:** Thanks for your advice. We have described the phylogenetic relationship analysis in materials and methods and **legend of new S11 Fig**, and the alignment of the proteins were shown in **supplemental file S1**.

15. S12 Fig. State how many experiments were performed and the consistency of the observations.

**Response:** Revised.

16. S13 Fig. State what is shown in Panel A (cDNA I assume).

**Response:** Revised.

17. S15 Fig C. Lane for L9 in western blot seems to be cropped from a different photo. Make a clear differentiation of photos of different blots.

**Response:** Thanks for your advice. We have made a clear differentiation of photos of different blots in **new S16C Fig**, and the original picture is shown as follow.

18. S3 Table. The IWGSC identifiers should be included for all putative interactors.

**Response:** We have added it in **new S3 Table**.
