# Supplementary material for: Glycine-serine-rich effector PstGSRE4 in Puccinia striiformis f. sp. tritici inhibits the activity of copper zinc superoxide dismutase to modulate immunity in wheat
Source: PLoS Pathog. 2022 Jul 26;18(7):e1010702. doi: 10.1371/journal.ppat.1010702 (PMC9321418; doi:10.1371/journal.ppat.1010702)
Supplement: Attachment — Submitted filename: Response to comments on PstGSRE4 msR2.docx [file ppat.1010702.s025.docx]

Re: PPATHOGENS-D-22-00159R1

Glycine-serine-rich effector PstGSRE4 in *Puccinia striiformis* f. sp. *tritici* inhibits the activity of copper zinc superoxide dismutase to modulate immunity in wheat

Dear Editors,

Thank you very much for handling our manuscript. We are grateful to the reviewers for their valuable comments, which certainly contribute to a significant improvement of the manuscript. In the revised manuscript, we have attempted to address all the comments from you and the reviewers. Below are our point-to-point responses to the comments from the reviewers. Thank you for your favorable consideration.

Best regards,

Jun Guo and Zhensheng Kang

College of Plant Protection

Northwest A&F University

Yangling, Shaanxi, 712100

PR China

Phone/Fax: +86 29 87082439

Email: guojunwgq@nwsuaf.edu.cn

**Reviewer 1 Comments for the Author**

In the revised manuscript, the authors have fully addressed all of my concerns. I believe the data to be convincing and sufficient to demonstrate the role of *Puccinia striiformis* f. sp. *tritici* (*Pst*) secretes glycine-serine-rich effector protein PstGSRE4 manipulate plant immunity through inhibition of the enzyme activity of wheat copper zinc superoxide dismutase TaCZSOD2, and reduction of H_2_O_2_ accumulation and HR areas to facilitate *Pst* infection. Clearly, many interesting avenues for follow up will emerge from this work from the authors as well as other groups in the field of plant-microbe interaction. I’m looking forward to seeing this work published.

**Response:** Thank you very much for your positive and constructive comments and suggestions on our manuscript.

**Reviewer 2 Comments for the Author**

The authors replied to all the comments I made on the previous version of the manuscript and I do not have further comments or questions.

**Response:** Thank you very much for your positive and constructive comments and suggestions on our manuscript.

**Reviewer 4 Comments for the Author**

This study reported a new glycine-serine-rich effector protein lacking the m9-like motif, PstGSRE4, which inhibits the enzyme activity of the wheat TaCZSOD2 to modulate ROS-associated defense responses. By inhibiting the enzyme activity of TaCZSOD2, PstGSRE4 reduces H_2_O_2_ accumulation and HR to facilitate *Puccinia striiformis* f. sp. *tritici* infection. These findings provide new insights into the molecular mechanisms of GSREs of phytopathogenic fungi in regulating plant immunity.

**Response:** Thank you very much for your positive and constructive comments and suggestions on our manuscript.

**Minor Issues:**

I have only one minor point that could be discussed. In addition to CZSOD2, the wheat (cultivar Suwon11) contains other three highly conversed TaCZSODs (TaCZSOD1, TaCZSOD3, TaCZSOD4). Since PstGSRE4 can directly interact with TaCZSOD2, and inhibit its’ activity. Why cannot TaCZSOD1/3/4 catalyze the production of H_2_O_2_ from O_2_^-^ during *Pst* infection？**Response:** Thank you for your suggestion. In Discussion Part we added as “In this study, we also identified other three highly conversed TaCZSODs, TaCZSOD1, TaCZSOD3, and TaCZSOD4. The enzyme activity of TaCZSOD1 has been also detected *in vitro* (Fig 6A and 6B), suggesting that TaCZSOD1 may catalyze the production of H_2_O_2_ from O_2_^-^ during *Pst* infection. Future work will be performed to investigate the functions of the three TaCZSODs during wheat-*Pst* interaction and reveal the molecular mechanisms of other *Pst* effectors targeting the CuZnSODs involved in wheat immunity.”
